# Fast and accurate dynamic estimation of field effectiveness of meningococcal vaccines

**DOI:** 10.1186/s12916-016-0642-2

**Published:** 2016-06-30

**Authors:** Lorenzo Argante, Michele Tizzoni, Duccio Medini

**Affiliations:** Department of Physics and INFN, University of Turin, via Giuria 1, Turin, 10125 Italy; ISI Foundation, via Alassio 11/C, Turin, 10126 Italy; GSK Vaccines, Siena, Italy

**Keywords:** Neisseria meningitidis, Vaccine effectiveness, Invasive meningococcal disease, Meningococcal carriage, Bexsero, Monte Carlo maximum likelihood, Computational dynamic models

## Abstract

**Background:**

Estimating the effectiveness of meningococcal vaccines with high accuracy and precision can be challenging due to the low incidence of the invasive disease, which ranges between 0.5 and 1 cases per 100,000 in Europe and North America. Vaccine effectiveness (VE) is usually estimated with a screening method that combines in one formula the proportion of meningococcal disease cases that have been vaccinated and the proportion of vaccinated in the overall population. Due to the small number of cases, initial point estimates are affected by large uncertainties and several years may be required to estimate VE with a small confidence interval.

**Methods:**

We used a Monte Carlo maximum likelihood (MCML) approach to estimate the effectiveness of meningococcal vaccines, based on stochastic simulations of a dynamic model for meningococcal transmission and vaccination. We calibrated the model to describe two immunization campaigns: the campaign against MenC in England and the Bexsero campaign that started in the UK in September 2015. First, the MCML method provided estimates for both the direct and indirect effects of the MenC vaccine that were validated against results published in the literature. Then, we assessed the performance of the MCML method in terms of time gain with respect to the screening method under different assumptions of VE for Bexsero.

**Results:**

MCML estimates of VE for the MenC immunization campaign are in good agreement with results based on the screening method and carriage studies, yet characterized by smaller confidence intervals and obtained using only incidence data collected within 2 years of scheduled vaccination. Also, we show that the MCML method could provide a fast and accurate estimate of the effectiveness of Bexsero, with a time gain, with respect to the screening method, that could range from 2 to 15 years, depending on the value of VE measured from field data.

**Conclusions:**

Results indicate that inference methods based on dynamic computational models can be successfully used to quantify in near real time the effectiveness of immunization campaigns against *Neisseria meningitidis*. Such an approach could represent an important tool to complement and support traditional observational studies, in the initial phase of a campaign.

**Electronic supplementary material:**

The online version of this article (doi:10.1186/s12916-016-0642-2) contains supplementary material, which is available to authorized users.

## Background

*Neisseria meningitidis* is an aerobic Gram-negative diplococcus that causes annually 1.2 million cases of meningitis and 135,000 deaths globally [1]. This human-restricted opportunistic pathogen is part of the commensal flora that colonizes the upper respiratory tract of healthy individuals. *N. meningitidis* strains are divided into 12 serogroups, on the basis of the immunochemistry of their capsular polysaccharides [2]. Serogroups A, B, C, W, Y, and X account for most of the invasive disease cases worldwide; serogroup B (MenB) is the leading cause of meningococcal meningitis in Europe (90 %), New Zealand (82 %), Australia (80 %), Argentina (67 %), Japan (57 %), and Canada (53 %) [3–11].

Invasive meningococcal disease (IMD) has the highest fatality rate among other vaccine-preventable diseases after rabies [12] (up to 40 % for meningococcal septicemia [13]), is easily misdiagnosed [14] and can cause death in 24 hours. Up to 20 % of survivors exhibit permanent lifelong disabilities, including brain damage, deafness, kidney failure, and limb amputation [15]. All age groups are susceptible to IMD. Infants, however, are 17 times more likely to develop disease compared to the general population [16]. A smaller peak of IMD incidence is also observed in adolescents and young adults. Notably, more than 10 % of the general population asymptomatically carries *N. meningitidis* [17], and in the first 30 years of life, each person is expected to become a carrier of the meningococcus 10 times [18]. The age distribution of asymptomatic carriage, however, is markedly different from that of IMD. Carriage in infants is low, and grows slowly up to approximately 10 % in pre-adolescents. A sharp increase of carriage prevalence is observed after 15 years of age, reaching 25 % or more at the age of 20, and then it decreases slowly to approximately 10 % in the elderly [19].

Due to the ambiguous initial clinical manifestations and to the extremely rapid development of disease, vaccination is the only broadly effective measure against IMD [15]. Vaccines based on serogroup A, C, W, and Y capsular polysaccharide conjugates have been licensed in many parts of the world [20–23]. Among others, a national immunization campaign that started in 1999 in the UK showed the high efficacy of meningococcal C (MenC) conjugate vaccines against serogroup-specific disease and carriage [21, 24–30]. Only very recently two broadly protective MenB vaccines were licensed, Bexsero (GSK) and Trumenba (Pfizer), both licensed in the USA for persons between 10 and 25 years of age. Bexsero was also licensed in Europe, Canada, Australia, and elsewhere for individuals from 2 months of age and older and is being used in a national immunization program for infants and children in the UK that started in September 2015 [31]. Both vaccines were licensed based on safety and immunogenicity data. Although strain coverage was also assessed for Bexsero worldwide through the Meningococcal Antigen Typing System [32], indicating strain coverage between 66 and 91 % worldwide [33], no formal proof of field effectiveness is available yet.

Mass immunization campaigns can be evaluated in the field by observational studies, monitoring the number of cases emerging in vaccinated and non-vaccinated cohorts [34]. A number, called *vaccine effectiveness* (VE), measures how much a vaccine is efficacious in reducing the incidence of a certain disease in groups of vaccinated, when compared to the incidence in subjects that have not received the vaccine [34].

Case–control studies would represent the first choice when estimating meningococcal VE, since cohort studies are not feasible due to the low disease incidence [34]. However, IMD incidence is so low that also the applicability of case–control studies is severely limited. A popular alternative to case–control studies is the screening method [35]. This method is a variant of the case–control methods where, instead of choosing one or more individual controls per case, the entire population at risk is used as a reference group [36]. The screening method estimates the VE using a mathematical formula (see Additional file 1 for details) that combines three basic quantities, measured at one point in time: the reported number of disease cases, the number of vaccinated among cases, and the proportion of the overall population under study that has been vaccinated [35]. Compared to other observational methods, screening is considerably less resource-intensive, because all the information needed is usually available from surveillance systems and does not require specific designs. Additionally, it has the great advantage of being rapid, and this is why it has been proposed as a first step to determine VE [36]. On the other hand, the screening method is known to lack precision, its estimates being very sensitive to errors in the input data [37, 38].

For meningococcal vaccines, the screening method is the main candidate to estimate VE. It has been employed to evaluate the 1999 MenC immunization campaign in England [25], and it will likely be used to estimate Bexsero effectiveness from field data. However, in this context its advantages are significantly reduced by the very low incidence of the disease, which will inevitably lengthen the time needed to obtain a precise VE estimate. Simple statistics arguments [35] allow us to predict the number of IMD cases that will be needed to estimate some hypothetical true value of VE with a desired precision, that is with a 95 % confidence interval (CI), possibly bounded not too far from the point estimate. Using available incidence data [39], cases can be converted into observation time. For instance, assuming that the expected IMD incidence in the UK will be the same as that experienced during 2012–2014, on average, and assuming a VE of 60 % or higher, at least 15 years will be needed to estimate effectiveness with a lower bound of the 95 % CI higher than 45 % (see Additional file 1 for details).

An important role in meningococcal vaccines is played by the ability to confer or not a mucosal immunity that can protect people from *N. meningitidis* nasopharynx colonization, therefore reducing asymptomatic transmissions of invasive strains. Here, we will refer to it as the *indirect* effect of the vaccine because it can provide protection also to unvaccinated individuals, conferring *herd immunity*. This mechanism can strongly impact the transmission dynamics and reduce the morbidity of pathogens like *N. meningitidis*, as has been observed and quantified for vaccines against MenC [28, 30, 40]. However, by employing notified IMD cases only, the screening method fails to evaluate the indirect VE, since it contributes to lower the IMD incidence both in vaccinated as well as in unvaccinated individuals (details in Additional file 1).

It is possible to evaluate VE by comparing the disease incidence among unvaccinated individuals only with the disease incidence in the same population, measured before the vaccination campaign [34]. This has been done for the MenC conjugate vaccines in England [29], providing evidence of a herd immunity effect, but with low precision and large fluctuations around the point estimate across age groups. A more robust way to evaluate the indirect effectiveness is by measuring the meningococcal carriage, instead of the invasive disease, at two points in time. For this purpose, large cross-sectional carriage studies have been conducted to quantify the meningococcal carriage in adolescents recruited in England before and after the vaccination campaign [28, 30]. However, such an approach is inevitably expensive and time-consuming.

The challenge of shortening the time needed to evaluate the direct VE and, at the same time, providing a tool to improve the evaluation of the indirect effectiveness using notified cases only, motivated our work. For this aim, we devised a VE estimation method based on a Monte Carlo maximum likelihood (MCML) inferential approach that combines stochastic simulations of a computational dynamic model for *N. meningitidis* asymptomatic transmission and real case notification data. More specifically, we built a discrete mechanistic model that stochastically reproduces the dynamics of *N. meningitidis* infection and vaccination at the population level. The model structure is based on previous works [18, 41–43] and explicitly takes into account both the direct and indirect effect of the vaccine as two free parameters. Unlike previous works, where modeling was used to forecast the impact of different vaccination strategies in England, generating scenarios of future disease incidence based on hypothetical values of VE, here we adopted a reverse approach: we inferred the most probable values of VE via likelihood maximization given the time series of disease cases notified before and after the campaign start, using a sequential Monte Carlo (SMC) method, also known as particle filtering [44].

The main goal of our study was to estimate the direct effectiveness of meningococcal vaccines with the same accuracy as the screening method but faster, aiming at a better precision in a shorter time. Furthermore, we wanted to show that our model-based approach allows for a simultaneous estimation of the indirect effectiveness, achieving similar results as carriage studies without using additional data beside IMD notifications and vaccine uptake.

## Methods

In our work, we focused on two different mass immunization campaigns against meningococcal disease in England: the MenC campaign that started in 1999 and the MenB campaign that started in September 2015. In the following, we describe the model’s definition, the data used to parameterize it, and the MCML inference method used to estimate the VE. Further details on the methods are reported in Additional file 1.

### Dynamic model of meningococcal transmission, carriage, and invasive disease

We defined a dynamical compartmental model that reproduces meningococcal carriage, transmission, invasive disease, and vaccination. In particular, we were interested in those characteristics that are relevant to the inference of (i) the VE in inducing protection from invasive disease, or *direct* effectiveness, and (ii) the VE in reducing the asymptomatic transmission, or *indirect* effectiveness. In the following, we will refer to them as VE_dir_ and VE_ind_, respectively.

**Fig. 1:**
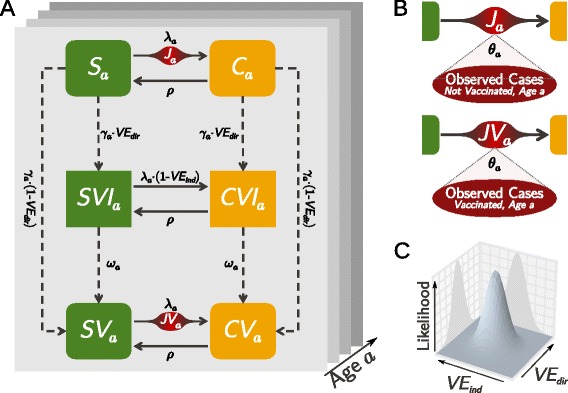
Schematic representation of the Monte Carlo maximum likelihood method to infer vaccine effectiveness. **a** An age-structured stochastic compartmental model reproduces the transmission and vaccination dynamics of *Neisseria meningitidis*. The population is stratified by age *a*, infection status (susceptible *S* or asymptomatic carrier *C*), and vaccination status (*V*), where the additional I indicates that the vaccination induced full immunity to the invasive disease. All compartments are subject to demographic transitions: birth, ageing, and death (not shown). At each time step, susceptibles are infected with probability *λ*_*a*_ and recovery from carriage to susceptible status happens with probability *ρ*. The force of infection *λ*_*a*_ is reduced by 1−VE_ind_ for those susceptibles who are successfully immunized (*SVI*). Individuals are vaccinated with probability *γ*_*a*_. A fraction VE_dir_ of them becomes fully immune to the invasive disease, while the remaining fraction is vaccinated but not immune. For a fraction *ω*_*a*_ of the immunes, the acquired immunity wanes after a time period *τ*_imm_. The outcome of the transmission model is the number of infections by age group in non-vaccinated and vaccinated compartments during an epidemiological year: *J*_*a*_ and *JV*_*a*_. We use these numbers in the observational disease model (**b**). Given the probability of developing the invasive disease for a single age group *θ*_*a*_, calibrated using data before vaccination, and given the number of cases observed in reality, we compute the likelihood function of VE_ind_ and VE_dir_ (**c**). The likelihood maximum will correspond to the best estimates of the two vaccine’s effectiveness. The two-dimensional likelihood is sliced in correspondence of its maximum to calculate confidence intervals around the best estimates

The compartmental structure of the model is shown in Fig. 1a. It is an age-structured Susceptible-Infected-Susceptible (SIS) model, similar to others already used in the literature for evaluating the impact of vaccination on *N. meningitidis* [41–43]. The entire population of England is divided into 160 age classes expressed in quarters of a year, plus one group of ≥40 years old. Individuals in a given age class are divided into two possible health statuses: susceptibles and asymptomatic carriers of *N. meningitidis*, represented by the symbols S and C, respectively. In addition, the population is divided into three vaccination statuses depending on the campaign schedule and the immunization outcome. Altogether the population of a given age class is stratified into six possible classes, namely susceptible and carrier that are: not vaccinated (*S* and *C*), vaccinated and immune (*SVI* and *CVI*), and vaccinated but not immune (*SV* and *CV*). By *immune*, we refer to the immunity against IMD provided by the vaccine. We model only those *N. meningitidis* strains or serogroups for which we want to assess the effectiveness of the vaccine under study. Therefore, no coinfection or competition between different meningococcal serogroups is possible.

Overall, the model is based on 966 compartments (i.e., the system’s variables), each one representing the number of individuals of a given age and infection and vaccination status at a given time. The system evolves under the dynamics defined by all the possible transitions between compartments. All variables are updated with a discrete time step corresponding to 3 months. At each time step, the following transitions are allowed:

- Birth. Newborns are added to the unvaccinated susceptibles of age 0, according to the natural birth rate.
- Ageing. Individuals in a given age group move to the next age group, if not already in the last age class.
- Death. Some individuals of a given age in every compartment die, according to the natural death rate.
- Infection. Susceptibles become carriers depending on the force of infection *λ*_*a*_(*t*). If vaccinated immune, the probability of being infected is reduced by a factor corresponding to 1−VE_ind_.
- Recovery. Carriers spontaneously lose the carrier status with frequency *ρ*, whose inverse 1/*ρ* is the average duration of carriage *τ*_car_.
- Progression to IMD. In rare cases, susceptible individuals develop IMD 2–10 days after meningococcal carriage acquisition [17, 45, 46]. We model progression to IMD as an event alternative to the asymptomatic infection. It can happen only immediately after transmission, with an age-dependent risk of IMD given infection, denoted as *θ*_*a*_(*t*).
- Vaccination. Unvaccinated individuals in cohorts targeted by vaccination are moved to the vaccinated compartments according to the vaccine schedule and the population coverage *γ*_*a*_. Only a fraction *VE*_dir_ of the vaccinated gains immunity to IMD.
- Waning of immunity. A fraction *ω*_*a*_ of the vaccinated and immune spontaneously move to the vaccinated non-immune compartment, after a time period *τ*_imm_ that depends on age and vaccine type (MenC or MenB vaccine).

The model is stochastic. The compartments and the transitions between them can be represented as a set of stochastic differential equations (see Additional file 1).

Once the model is parameterized to reproduce the epidemiology of meningococcal disease in the absence of vaccination campaigns, the effectiveness is inferred via MCML by means of two parameters, namely the VE against invasive disease VE_dir_ and against carriage acquisition VE_ind_. Both are bounded between zero and one, but they act in very different ways. At each time step after the start of the vaccination campaign, VE_dir_ represents the fraction of vaccinated individuals who successfully gain vaccine-induced protection against IMD. Those individuals are, therefore, immune and cannot develop the IMD. The remaining fraction (1−VE_dir_) of individuals go into the not-immune compartments and can develop the disease as if they were not vaccinated at all. In this sense, our modeling approach uses an all-or-nothing vaccine [34].

On the other hand, VE_ind_ is a parameter that multiplies only the force of infection experienced by vaccinated and immune compartments, thus reducing the probability of meningococcal colonization, i.e., the transition *SVI* →*CVI*. It represents the ability gained by vaccinated subjects for developing a certain level of protection against *N. meningitidis* acquisition.

The parameters VE_ind_ and VE_dir_ are in fact *inputs* of the transmission model, which is otherwise completely specified by the other parameters and the initial compartment population. The outputs of the transmission model are the numbers of infection events per time step that can generate IMD cases (transitions *S* →*C* and *SV* →*CV*). We call these numbers *J*_*a*_(*t*) and *JV*_*a*_(*t*), respectively, for non-vaccinated and vaccinated of age *a*. They will be used to infer which values of VE will most likely lead to the reported time series of IMD cases. It is important to note that the number of infections experienced by immune individuals is not collected because in the model formulation, those infections cannot lead to IMD cases.

In the model, diseased individuals are not explicitly treated as a compartment. The risks *θ*_*a*_ are used to link the transmission model, whose outcomes are *J*_*a*_(*t*) and *JV*_*a*_(*t*), quantitatively to the number of IMD cases reported by the surveillance. The progression-to-disease process is, in fact, treated as an observational process (see Fig. 1b and Additional file 1 for more details). The number of lab-confirmed IMD cases of age *a* observed during a certain time period *τ*, $D_{a}^{\text {obs}}(\tau)$, is modeled to follow a binomial distribution:

1 $$\begin{array}{*{20}l}  D_{a}^{\text{obs}}(\tau) & \sim \operatorname{bin} (J_{a}(\tau),\theta_{a}),  \\ {DV}_{a}^{\text{obs}}(\tau) & \sim \operatorname{bin}({JV}_{a}(\tau),\theta_{a}), \end{array} $$

where the right-hand side represents the probability that $D^{\text {obs}}_{a,v}(y)$ IMD cases emerge from *J*_*a*,*v*_(*y*) infection events, when the probability of an IMD given infection is *θ*_*a*_.

### Model parameterization

All the parameters, except for VE, are quantified by calibrating the model in the absence of immunization campaigns on demographic, carriage, and disease data, or by integrating estimates reported in the literature (see Table 1 for a full list of parameters and their values).

**Table 1:** Parameter values of the dynamic model

	Description	Value	Source
*α*	Yearly crude birthrates	Between 11.3 and 13.0 per 1000 population, variable by year	[47]
*μ*_*a*_	Yearly mortality rates	Variable by age (Additional file 1: Figure S2)	[47]
*τ*_car_	Duration of carriage (1/*ρ*)	6 [3–9] months	[41, 43, 49]
*β*_*a*_	Quarterly rates of infection given contacts with carriers	Variable by age (Additional file 1: Figure S4)	Fitted
$m_{aa'}\phantom {\dot {i}\!}$	Daily contact rates matrix	Variable by age of subject and contact (Additional file 1: Figure S3)	[48]
*θ*_*a*_	Quarterly risks of disease given infection	Variable by age (Additional file 1: Figure S5)	Fitted
*γ*_*a*_	Quarterly fraction of population vaccinated	MenB: 95 % of 1 year olds MenC: variable by age and schedule	MenB: assumed MenC: [52]
*τ*_imm,*a*_	Duration of immunity to disease	MenB: 18 months MenC: variable by age (15 [9–24] mths, 5 years, 10 years)	MenB: assumed MenC: [25, 41]
*ω*_*a*_	Waning of immunity fraction	MenB: 100 % MenC: 58.4 %	MenB: assumed MenC: [26]

The model reproduces the English demographic structure and its temporal evolution, according to population estimates and annual birth and death rates, as reported by the UK Office of National Statistics [47].

Before vaccination, the model is parameterized by assuming the carriage prevalence to be temporally stable across age groups [19], thus we constrain the model’s equations to keep the carriage by age constant at every time step. The carriage prevalence of C and B serogroups by age class is assumed to follow the average overall meningococcal carriage age pattern reported in European studies [19], after weighting on the specific fraction of C or B serogroups reported before the beginning of the campaign [30]. The number of infections per time step depends on the force of infection *λ*, which is proportional to the fraction of carriers among contacts:

2 $$\begin{array}{*{20}l} {}\lambda_{a}(t) &= \beta_{a} \sum_{a'} m_{aa'} \frac{\text{carrier population of age } a' \text{ at time } t}{\text{total population of age } a' \text{at time } t}. \end{array} $$

The symbol $\phantom {\dot {i}\!}m_{aa'}$ denotes the reported number of daily contacts between persons of age *a* and *a*^′^, and it is assumed to follow contact patterns observed in Great Britain [48]. The carriage acquisition rate *β*_*a*_ is a free parameter to be calibrated against serogroup-specific carriage data (see Additional file 1 for details).

Infected individuals lose their carrier status with probability *ρ* per time step and can be infected again. We set *ρ*=1/*τ*_car_ with *τ*_car_ representing the average duration of carriage. In our base case, we assumed *τ*_car_ to be equal to 6 months and independent from the serogroup under study. For a sensitivity analysis, we tested values between 3 and 9 months as reported in the literature [18, 41, 43, 45, 49–51] (see Additional file 1 for details).

During the immunization campaign, a fraction *γ*_*a*_ of non-vaccinated individuals belonging to cohorts scheduled for vaccination moves to the vaccinated compartments. Only individuals who have received all the recommended doses are taken into account and they are moved according to the approximate dates of injections, for both routine and catch-up implementation. The MenC campaign coverage data have been published [25, 41, 52]. For Bexsero, we use expected coverage data, following previous works [43, 53].

The vaccine confers protection for a limited time and the duration of vaccine-induced immunity *τ*_imm_ depends on the specific vaccine and the age of the vaccinated cohort. For the MenC campaign, the protection is set to last 15 months for the routine vaccination, 5 years for children vaccinated at 12 months, and 10 years for all those vaccinated at older ages [41]. For the MenB campaign, we assume infants to be routinely vaccinated at 2, 4, and 12 months, as announced by Public Health England (PHE) [54]. Estimates of the duration of protection for Bexsero are not available yet [55]. We assume the vaccine-induced protection to last at least 18 months after the booster dose, in line with previous works [43].

Meningococcal disease in the UK is primarily endemic, with episodic hyperendemic waves [45, 56, 57]. We calibrate the age-dependent risks of IMD given infection to be

3 $$\begin{array}{*{20}l} \theta_{a} &= \frac{D^{\text{exp}}_{a}}{J^{\text{exp}}_{a}}, \end{array} $$

where the numbers $D^{\text {exp}}_{a}$ are occurrences of IMD cases and the $J^{\text {exp}}_{a}$ are the numbers of infection events produced by the model, both *expected* to emerge during a time step when no vaccination campaign is implemented. The *D*^exp^ are the yearly average numbers of laboratory-confirmed IMD cases by age, reported during the last 1–3 epidemiological years before the vaccination campaign. The numbers $J^{\text {exp}}_{a}$ are calculated from the infection term of the model’s equations set at the initial time step (see Additional file 1). The risks *θ*_*a*_ of IMD given infection by age are fixed, implying the assumption that the pathogenicity of meningococci would remain constant in the absence of immunization campaigns.

### Inference of VE

The MCML procedure that we use to infer the parameters VE_dir_ and VE_ind_ is based on a SMC approach, also known as particle filtering [44]. This method was first introduced in epidemiology to estimate the environmental contribution to cholera transmission [58], and then applied to a variety of epidemic models [59–61]. Here, the likelihood of the combination of *VE*s is calculated given empirical observations, which are time series of the number of cases by age group notified during each epidemiological year after vaccination start. The observables are connected to the above-described transmission model by means of the risks *θ*_*a*_.

Consider $D_{a,v}^{\text {obs}}(1:Y)$, the time series of notified cases per epidemiological year *y*=1,…,*Y*, age *a*, and vaccination status *v* (*v*=0 denotes unvaccinated and *v*=1 vaccinated). The likelihood of VE_dir_ and VE_ind_ is

$$ {{\begin{aligned} {}\mathcal{L}({VE}_{\text{dir}},{VE}_{\text{ind}}) & = \prod_{a,v} P(D^{\text{obs}}_{a,v}(1:Y)|{VE}_{\text{dir}},{VE}_{\text{ind}},H) \\ & = \prod_{a,v} \prod_{y=1}^{Y} P(D^{\text{obs}}_{a,v}(y)|D^{\text{obs}}_{a,v}(1:y-1),{VE}_{\text{dir}},{VE}_{\text{ind}},H). \end{aligned}}}  $$

*P* is the probability of observing cases given the transmission model (denoted as *H*), the history of observed cases, and the *VE* values. It is calculated as:

4 $$\begin{array}{*{20}l} {} P(D^{\text{obs}}_{a,v}(y)|D^{\text{obs}}_{a,v}(1:y\,-\,1),{VE}_{\text{dir}},{VE}_{\text{ind}},H) \,=\, \text{bin}(J_{a,v}(y),\theta_{a}). \end{array} $$

The particle filtering procedure allows us to sample the trajectories probabilistically in the space of the *J*, taking into account the history of cases and the model structure.

The best estimates of VE_dir_ and VE_ind_ correspond to the maximum of the likelihood. Likelihood profiling allows to calculate 95 % CIs around it.

### MCML method settings for the MenC vaccination in England

In the first part of our work, we simulated the mass immunization campaign against MenC disease that started in England at the end of 1999. To calibrate the model, England’s population is initially set to mid-1998 estimates. The age-dependent distribution of MenC IMD cases $D^{\text {exp}}_{a}$ used to calibrate the method is the one reported during 1998 and the first half of 1999, then normalized to reproduce, on average, the total number of cases notified during the epidemiological year 1998/99. As a sensitivity analysis, we tested different $D^{\text {exp}}_{a}$ by varying the average total number of cases generated by the model, while keeping a constant age distribution (see Additional file 1: Section 5). We normalized the carriage prevalence distribution by age to reproduce the prevalence of serogroup C samples observed in carriage studies before 1999 [25]. The SMC algorithm was run using 1000 particles and varying the values of VE_dir_ and VE_ind_ between 0 % and 100 % with steps of 0.1 % to build the yearly likelihood surfaces.

### Simulation of MenB vaccine campaign in England and MCML settings

In the second part of our work, we simulated the mass immunization campaign against MenB disease that started in England in September 2015. The initial population of England employed for calibration is the one estimated in 2014. To calculate the expected occurrences of MenB cases $D^{\text {exp}}_{a}$, we used the age-dependent distribution of MenB IMD cases reported during the epidemiological year 2007/08, then normalized to produce, on average, the total number of cases notified every year from mid-2012 to mid-2015. The carriage prevalence distribution by age was normalized to reproduce the prevalence of serogroup B samples observed in carriage studies [30].

In this scenario, disease case occurrences after the campaign start $D^{\text {obs}}_{a,v}$ are not available yet. To test our approach, we used the model in a generative way, thus simulating future incidence scenarios with constant values of VE_dir_ and VE_ind_, as usually done to predict the impact or the cost-effectiveness of vaccination campaigns [41, 43, 62]. In detail, we assumed VE_ind_ to be always equal to 0 %, to be as conservative as possible, since reliable estimates of the indirect effect of Bexsero are not available. Then, we assumed four possible values of VE_dir_: 60 %, 70 %, 80 %, and 90 %. For each of the four scenarios, we ran the model 10,000 times, thus producing 10,000 trajectories in the space of the MenB disease case occurrences, stratified by age and vaccination status. Then, we selected the median trajectory to be the reference time series of synthetic MenB cases observed after vaccination.

Given the simulated IMD incidence curve, we used the MCML to estimate the most likely value of VE_dir_ and the corresponding CIs at different years, by assuming a constant VE_ind_. In each scenario, the SMC algorithm was run using 1000 particles and varying the values of VE_dir_ between 0 % and 100 % with steps of 0.1 % to build the yearly likelihood curves.

Finally, we compared the results obtained with the MCML method against the screening method’s sample size analysis [35]. In particular, for eight consecutive years after the start of the vaccination, we compared the MCML point estimate of VE_dir_ and its CI against the CI provided by the screening method, for the same value of VE_dir_. We estimated the screening method’s CIs by converting the required sample of IMD cases given by the Farrington formula [35] to the time needed to observe such cases (see Additional file 1 for details). Here, we calculated that the baseline incidence of IMD would be 70 cases a year in the fully immunized cohort (12–23 months old), if no vaccination is implemented.

## Results

The four main results that we achieved using the MCML method are:

1. The underlying calibration of the dynamic model allows us to reproduce realistically the epidemiology of meningococcal disease in England.
2. The MCML method allows us to estimate both direct and indirect VE using case notification data.
3. It provides realistic estimates of the VE compared to observational methods.
4. It is faster than the screening method in reaching the same precision around the point estimate of VE.

Here, we discuss these in detail.

### The dynamic model is calibrated to reproduce meningococcal epidemiology realistically

We calibrated the model for simulating two different vaccination campaigns in England, against the serogroup C and B meningococcal disease, as described in “Methods”. In both cases, we tested the calibration by running the model for 50 years under the basic transmission dynamics without any vaccination campaign.

The model reproduces a realistic demographic evolution of the population in England, with fluctuations due to population differences across age groups (see Additional file 1: Figure S6). The carriage prevalence remains constant for all age groups (see Additional file 1: Figure S7), indicating that the model has been adequately formulated and calibrated to reproduce the temporal stability of asymptomatic carriage [19]. The model reproduces the endemicity of the IMD, and the age pattern of cases remains roughly constant from year to year, as reported in the literature [63], with fluctuations due only to the demographic shifts (see Additional file 1: Figure S7).

### The MCML method estimates both direct and indirect effectiveness

In November 1999, a large mass immunization campaign against MenC disease started in England and Wales, consisting of a routine vaccination of infants and a catch-up vaccination targeting people up to 18 years old [52]. The overall MenC IMD incidence fell by about 80 % in the following 2 years. This achievement was accomplished thanks to the high effectiveness of the vaccine, which was found to provide both direct and indirect protection [28].

**Fig. 2:**
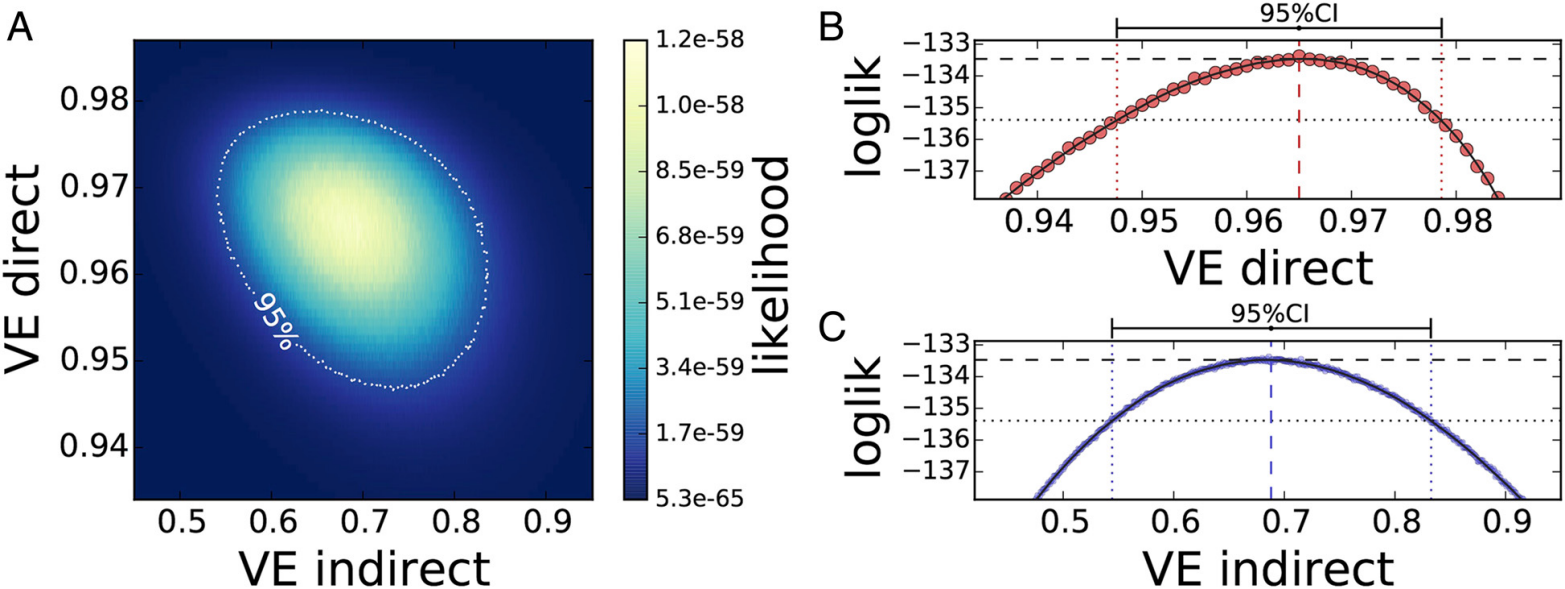
Maximum likelihood profiles for direct and indirect effectiveness against MenC. **a** The likelihood function of VE_dir_ (*y*-axis) and VE_ind_ (*x*-axis) calculated using IMD cases reported during the first two full epidemiological years of the MenC immunization campaign in England (cases notified from June 2000 to June 2002). The maximum is colored in *light yellow*, and unlikely values of VE are in *dark blue*. The 95 % CI is included inside the *white dotted line*. The log-likelihood function is sliced around its maximum, as a function of VE_dir_ (**b**) and VE_ind_ (**c**). The *solid line* interpolates the discrete values of the log-likelihood to identify the 95 % CLs, as indicated by the *vertical dotted lines*. *CI* confidence interval, *CL* confidence limit, *IMD* invasive meningococcal disease, *loglik* log-likelihood, *MenC* serogroup C meningococcal

After calibrating the model to reproduce the vaccination schedule of the MenC campaign (see “Methods”), we used the time series of IMD cases by age groups notified to PHE since mid-2000 to infer the values of VE_dir_ and VE_ind_ via MCML. Figure 2a shows the two-dimensional likelihood function of the VE values obtained using the cases reported between mid-2000 and mid-2002 (the first two full epidemiological years after the campaign started).

The likelihood function displays a unique and well-defined maximum in both dimensions, indicating that both the direct effectiveness and indirect effectiveness are identifiable parameters of the model under the conditions investigated and can be co-estimated by MCML. The 95 % CI of the most likely values are included in the white dotted line. Fig. 2b, c shows the logarithm of the unidimensional likelihoods of Fig. 2a, as a function of VE_dir_ and VE_ind_, respectively, obtained by slicing the log-likelihood surface in correspondence of the maximum. The best estimates of VE_dir_ and VE_ind_ and the corresponding 95 % CIs are shown as vertical dashed lines.

Here, we see that the model’s structure allows us to disentangle the two effects of the vaccine, which concur with the global decline of cases. Remarkably, the inferential MCML method can provide an estimate of the two parameters independently and very parsimoniously in terms of field data, comparable with the screening method.

### The MCML method accurately estimates MenC VE

To assess the quality of the MCML method, we compared the best estimates of VE_dir_ and VE_ind_ with a number of independent measures of the same quantities reported in the literature.

**Fig. 3:**
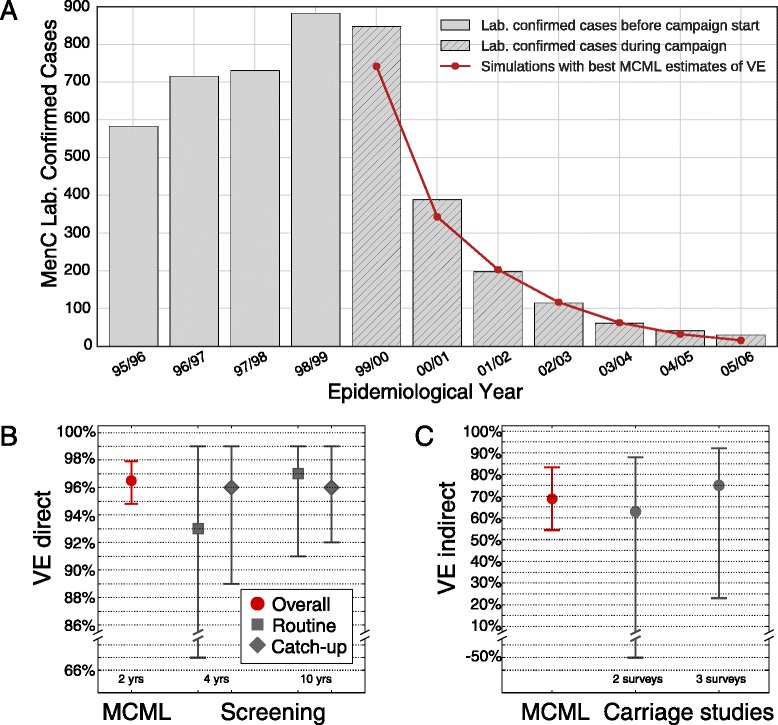
Vaccine effectiveness of MenC vaccine in England. Number of serogroup C disease cases notified by Public Health England (PHE) during the epidemiological years between 1995 and 2006. The vaccination campaign started in November 1999 (**a**). The *red solid line* represents the average number of IMD cases generated by the model, with the best estimates of VE_dir_ and VE_ind_. The MCML best estimate of vaccine effectiveness against disease VE_dir_ after 2 years of observed disease cases in the cohorts targeted by routine or catch-up vaccination is 96.5 % [94.8 %, 97.9 %] (**b**, in *red*). *Gray symbols* in (**b**) indicate the results obtained after 4 and 10 years by observational studies [25, 26] using the screening method. The MCML method simultaneously estimates the indirect vaccine effectiveness VE_ind_ (**c**, in *red*) with a best estimate equal to VE_ind_=68.8 % (95 % CI = [54.4 %, 83.3 %]), based on PHE notified cases. This value is compared to best estimates based on carriage studies [28, 30] (**c**, in *dark gray*). *IMD* invasive meningococcal disease, *MCML* Monte Carlo maximum likelihood, *MenC* serogroup C meningococcal

Studies based on the screening method previously assessed the direct effectiveness using field data [25, 26]. After 4 years of MenC vaccination, the direct VE was estimated to be 93 % (CI = [67 %, 99 %]) for the routine vaccination and 96 % (CI = [89 %, 99 %]) for the catch-up [25], as reported in Fig. 3b with gray symbols.

More precise estimates were published after monitoring the occurrence of MenC disease cases for 10 years [26]. The catch-up vaccination was confirmed to be 96 % effective (CI = [92 %, 99 %]). The effectiveness of routine vaccination was slightly higher than previous estimates: 97 % (CI = [91 %, 99 %]).

Considering vaccine uptake statistics and IMD case reports stratified by age and vaccinal status from mid-2000 to mid-2002, the MCML method estimated the direct effectiveness to be VE_dir_=96.5 % (red symbols in Fig. 3b), in good agreement with the value found by Campbell et al. [26]. The MCML method reached a higher precision than the screening method (95 % CI = [94.8 %, 97.9 %]), yet required a shorter observation period.

The indirect effectiveness of the MenC vaccine was estimated a few years after the campaign start, based on carriage studies. Figure 3c shows the best estimates and 95 % CIs of the indirect effectiveness, represented as gray dots and whiskers, published in 2002 [28] and 2008 [30]. Comparing the carriage prevalence reported by the 1999 survey and the one observed 1 year later, VE_ind_ was estimated to be 63 % (CI = [ −50 %, 80 %]) [28]. After collecting additional data on the carriage prevalence with a third survey in 2002, VE_ind_ was 75 % (CI = [23 %, 92 %]) [30]. As described in the previous section, the MCML also provided an estimate for VE_ind_, based on IMD incidence data only. Figure 3c shows in red the best MCML estimate, corresponding to VE_ind_=68.8 % (95 % CI = [54.4 %, 83.3 %]). Also in this case, the value is in good agreement with those published by Maiden and collaborators and the uncertainty around the point estimate is much smaller.

### MCML is more precise than the screening method

Finally, we assessed the applicability and the expected performance of the MCML method in estimating the VE of the 2015 MenB campaign in England. As described in “Methods”, here we assumed that Bexsero can provide only direct protection against IMD.

**Fig. 4:**
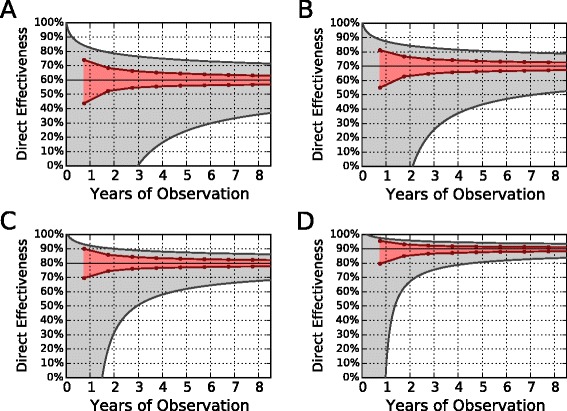
Comparing MCML and the screening method for Bexsero. Confidence intervals around four hypothetical values of VE_dir_=60 % (**a**), 70 % (**b**), 80 % (**c**), and 90 % (**d**) for the Bexsero immunization campaign in England, as a function of years required to obtain the precision. The *shaded red area* indicates the predicted CI produced by the MCML method. The *shaded gray area* indicates the predicted CI obtained with the screening method power analysis. *CI* confidence interval, *MCML* Monte Carlo maximum likelihood

Figure 4 shows the results of four hypothetical scenarios, where we considered different values of the best estimate for VE_dir_. Each panel shows the 95 % CI around the best estimate of VE_dir_ computed with the two methods, the screening method (in gray) and the MCML method (in red), and plotted against the observational time expressed in years after the campaign start.

In Fig. 4a, we assumed VE_dir_=60 %. The gray area bounded by solid lines indicates the extension of the 95 % confidence limits (CL), calculated using the Farrington formula. The higher 95 % CL converges faster than the lower one to the best estimate. The lower CL requires about 3 years just to reach 0 % effectiveness. To get a fairly precise estimate, we would need to wait longer. If we consider as *sufficiently precise* a lower 95 % CL not further than 15 % from the point estimate, thus above VE_dir_=45 %, we must wait 15.6 years from the beginning of the campaign. The shaded red area displays the 95 % CI for VE_dir_=60 % obtained with the MCML method. Here, the red dots indicate the CLs that we could obtain at mid-2016 (9 months of vaccination), mid-2017 (1 year and 9 months of vaccination), mid-2018, and so on. For all estimates, the CLs of the MCML method are well inside the interval of the screening method. Compared to the screening method, the MCML lower 95 % CLs are about 20-fold faster in getting close to the point estimate: after 9 months of observation, the MCML can provide acceptable CIs, with the CLs about 15 % distant from 60 %.

Figure 4b–d show the same comparison between the two methods assuming a best estimate of VE_dir_=70 %, 80 %, and 90 %, respectively. As expected, as the effectiveness increases, the time required to estimate VE_dir_ precisely becomes shorter for all methods. However, in all panels the red area identifying the CLs of the MCML method always falls well inside the gray area, indicating that the CLs of MCML converge faster than the screening method to the point estimate. As the effectiveness increases to 90 %, the difference between the higher 95 % CLs gets smaller. On the other hand, the lower 95 % CLs remain far enough away for there to be a substantial advantage with the MCML method. For instance, for VE _best_ = 90 % (Fig. 4d), the screening lower 95 % CL reaches 80 % in about 4.6 years. In contrast, the MCML lower 95 % CL reaches 80 % in only 9 months, while in the same 9 months, the screening method is not expected to get over 0 %.

## Discussion

The use of MCML methods to infer relevant epidemiological parameters from dynamic epidemic models is becoming more and more important. There are several examples in the literature of similar approaches aimed at characterizing epidemiological quantities that would not be accessible from incidence or prevalence data only, such as the worldwide transmission potential of pandemic flu [64], the environmental contribution to cholera transmission [59], or the interaction between influenza and pneumonia [61]. Here, we have shown that a MCML approach can be useful also in estimating unknown quantities, such as the VE, which could be easily quantified with incidence data as well, but over a longer time frame and with higher uncertainty, given the specific characteristics of meningococcal disease. Overall, our results suggest that a MCML approach to estimate the VE could be generally relevant for vaccines preventing low-incidence diseases, whose effectiveness may need several years to be estimated with enough precision by observational methods. From this perspective, the methodological framework presented in this work is rather general and could be extended in principle to different diseases and immunization scenarios where the limitations of the screening method are known to be relevant [37].

It is important to notice, though, that our approach relies considerably on the integration of a large body of knowledge about the transmission and colonization mechanisms, the emergence of the invasive disease, and the global epidemiology of *N. meningitidis*. Any extension to different diseases would necessarily require the availability of similar or better knowledge for the pathogen of interest.

For the same reason, our study carries some limitations. The power of the MCML method in providing a fast, accurate, and more precise estimate of VE than the screening method can be ascribed to two main elements:

1. The MCML method fits the whole time series of disease cases before and after vaccination, while estimates by the screening method are based on a single data point in time.
2. The dynamic model must be calibrated on epidemiological data by incorporating a large number of assumptions about the population structure, the means of transmission, and the incidence and prevalence of the disease.

While the first element does not carry significant limitations, the calibration on meningococcal epidemiology before the vaccination campaign may affect the MCML results. Specifically, the initial calibration relies on two assumptions: the carriage prevalence across age groups is forced to be stable from year to year and the risk of IMD given transmission by age and per time step is fixed to reproduce the disease endemicity. Despite the abundance of information on meningococcal disease, the relation between disease incidence and carriage prevalence is unclear. In particular, in the literature there are no clinical studies broadly assessing acquisition rates of meningococcal carriage in different age classes. Only a very recent work [65] provided an estimate of acquisition rates, measured by aggregating subjects between 10 and 25 years old. To fill this gap, we had to calculate indirectly the transmission rates *β*_*a*_, by combining available information on the carriage prevalence by serogroup and the duration of carriage *τ*_car_. Different combinations of these parameters may lead to the same transmission rates, but to different MCML estimates of VE. For instance, assuming a shorter duration of carriage is equivalent to considering a smaller fraction of carriage due to a single serogroup.

We tested the sensitivity of our VE estimates on variations of *τ*_car_, and we found that estimates of VE_dir_ are fairly unaffected by the assumed value of *τ*_car_. In contrast, estimates of VE_ind_ are sensitive to the duration of carriage: assuming a longer duration of carriage would lead to higher estimates of the indirect effectiveness. In Additional file 1: Figure S9, we show and discuss in detail how estimates of VE_ind_ and VE_dir_ for the MenC vaccine vary as *τ*_car_ is varied between 4 and 8 months, as well as how they evolve in time, with an increasing number of data points available for inference.

In general, there is not a strong consensus around the average duration of asymptomatic carriage, whose assessment requires large longitudinal carriage studies. In a recent review [45], the authors indicated 5–6 months as a reasonable value for *τ*_car_, and the most recent modeling efforts rely on this value [43, 62]. We, therefore, chose *τ*_car_=6 months as our baseline case. Retrospectively, this appears to be a good choice since it produces the best estimates of VE _ind_ that are in agreement with those that have been published in the literature (Fig. 3). Further applications of our approach would need to identify carefully the most plausible values of *τ*_car_; however, it is important to note that changes in *τ*_car_ do not affect the results presented here for Bexsero, since we only estimate its direct effectiveness. More importantly, additional clinical studies aimed at measuring acquisition rates of meningococcal carriage would be extremely helpful in removing such uncertainties.

We also assumed that the risks of invasive disease given transmission *θ*_*a*_ are constant over time. This assumption, combined with the temporal stability of carriage, reproduces the observed endemicity of meningococcal disease. Therefore, the model does not capture long-term trends in the incidence of disease, such as the decline of invasive disease observed in MenB or the rise of MenC cases observed just before vaccination. The mechanisms behind such trends are not completely understood so we do not try to model them; however, this assumption could lead to over- or underestimating the VE. To address this point, we tested whether, in the MenC setting, the inference of VE is affected when the model is calibrated assuming different values of *θ*, that is, different values of the total number of IMD cases occurring every year. As reported in Additional file 1, we found that estimates of the direct effectiveness are robust against variations in the assumed number of IMD cases generated by the model. Even by varying the number of expected IMD cases in 1999 from 600 to 1000, against a baseline value of 869, the MCML estimate of VE_dir_ is basically unaffected. On the other hand, estimates of VE_ind_ may vary significantly, as reported in Additional file 1: Table S4.

In summary, the MCML method is based on stochastic simulations of a dynamical compartmental model that describes the transmission of *N. meningitidis* in the population. The model is calibrated on epidemic data before vaccination, and, once the model is calibrated, the parameters VE_dir_ and VE_ind_ are uniquely identified via MCML. We carefully considered the impact of all the assumptions we made to parameterize the model fully before vaccination and found that, overall, estimates of VE_dir_ are very robust to changes in the initial calibration assumptions, thus supporting the validity of our method as a good candidate for an initial assessment of VE from field data. On the other hand, we found that estimates of VE_ind_ are significantly affected by the specific choice of parameterization, which, therefore, should be carefully assessed before evaluating the indirect effect of a meningococcal vaccine.

Finally, we tested the robustness of the MCML estimates against changes in the duration of vaccine-induced protection *τ*_imm_ for the routine vaccination in the MenC campaign. By varying the duration of immunity between 9 months and 24 months, we found that estimates of both VE_dir_ and VE_ind_ are not significantly affected by this parameter, as shown in Additional file 1: Figure S10.

Another important assumption we made is that a single strain model can be used to describe the transmission dynamics of different meningococcal serogroups (C and B), thus neglecting the interaction between serogroups. Although modeling this interaction could explain more realistically the age-dependent risk of disease given infection [42], and it might impact our estimates of VE [66], it comes at the cost of introducing several unknown parameters that must be estimated with the little empirical knowledge available. Here, we opted for a more parsimonious approach in terms of parameterization aiming at reducing all possible model uncertainties, thus minimizing the uncertainty on the VE. Moreover, the interaction between serogroups is expected to play a relevant role on long time scales, such as decades or hundreds of years [66], while we are interested only in the dynamics of the first few years after vaccine introduction.

## Conclusions

In this work, we have shown how a MCML method, combining stochastic simulations of a dynamic model and field data, can provide a fast and accurate estimate of the effectiveness of vaccines against meningococcal disease, by integrating epidemiological and demographic knowledge into an inferential framework.

We have retrospectively tested the MCML method on the MenC vaccination campaign that started in 1999 in England and found estimates of the VE that are in good agreement with those obtained with the classic screening method. In this context, we showed three main advantages of the MCML method: the shorter time required to obtain estimates, the higher precision in terms of CIs, and the ability to quantify both the direct and indirect effect of the vaccine, based on disease incidence data only.

Finally, we have shown how these advantages could have a high practical importance in estimating the effectiveness of Bexsero, a multicomponent vaccine that is currently being administered in a mass immunization campaign that started in England in September 2015. It will be important to test the MCML method for the ongoing campaign as soon as data on IMD incidence are available. This approach could provide an initial estimate of the VE, which, along with traditional observational methods, could support the work of public health officials. Furthermore, Bexsero is made with four antigenic components. The method presented in this work could be extended by considering the four antigens as individual strains to assess how each component contributes to the overall VE.

## Abbreviations

C, carrier not vaccinated; CI, confidence interval; CL, confidence limit; CV, carrier vaccinated but not immune; CVI, carrier vaccinated and immune; IMD, invasive meningococcal disease; MCML, Monte Carlo maximum likelihood; MenB, serogroup B meningococcal; MenC, serogroup C meningococcal; PHE, Public Health England; S, susceptible not vaccinated; SIS, Susceptible-Infected-Susceptible; SMC, sequential Monte Carlo; SV, susceptible vaccinated but not immune; SVI, susceptible vaccinated and immune; VE, vaccine effectiveness

## Additional files

Additional file 1 Supplementary information. Supplementary information on the screening method, the model’s formulation and sensitivity analysis. (2360 KB PDF)

Additional file 2 England population data. England population in 1999 and 2014 by age. (6 KB XLSX)

Additional file 3 Meningococcal carriage data. Distribution of meningococcal carriage by age in Europe. (6 KB XLSX)
