# Supplementary material for: Fast and accurate dynamic estimation of field effectiveness of meningococcal vaccines
Source: BMC Med. 2016 Jun 30;14:98. doi: 10.1186/s12916-016-0642-2 (PMC4929770; doi:10.1186/s12916-016-0642-2)
Supplement: Additional file 1 — Supplementary information. Supplementary information on the screening method, the model’s formulation and sensitivity analysis. (2360 KB PDF) [file 12916_2016_642_MOESM1_ESM.pdf]

Fast and accurate dynamic estimation of field effectiveness for meningococcal vaccines
Supplementary Information

Lorenzo Argante, Michele Tizzoni, Duccio Medini

April 29, 2016

Here, we provide all the details on the screening method to estimate the vaccine effectiveness, the dynamic model definitions, the results of the model calibration, the Monte Carlo Maximum Likelihood method to estimate VE, and the sensitivity analysis on the model parameters.

S1 Estimating the vaccine effectiveness with the screening method

Vaccines are usually evaluated by means of a number, called *vaccine effectiveness* (VE), calculated as one minus a measure of relative risk (RR) [1]:

$$\mathrm{VE} = 1 - \mathrm{RR}. \tag{S1}$$

The relative risk is a ratio of two risks: the risk that a certain event occurs in a group of people divided by the risk that the same event happens in a second distinct group of people. In this context, the two groups are composed by vaccinated and unvaccinated individuals respectively, while the event of interest is some observable related to a particular infectious disease, for instance clinical observation of illness, possibly supported by biological tests to confirm the infectious agent, or sampling of pathogens in asymptomatic individuals. The risks are probabilities, often approximated by rates.

In case-control studies [2], cases are ascertained, and their vaccination statuses are retrospectively observed. Then, a random sample of the same population to which the cases belong (the controls) is drawn to give information about the proportion of vaccinated persons. Thus, cases in case-control studies represent a sampling fraction of all cases and controls represent a different sampling fraction of the healthy susceptible population. In order to avoid possible potential biases, the populations should share epidemiologically relevant characteristics, such as sex, age or geographical area, depending on the disease

under study. The ratio of vaccinated over unvaccinated cases is divided by the ratio of vaccinated over unvaccinated controls. This is not a ratio of two risks, but rather an *odds ratio* (OR). However, for rare disease, OR $\simeq$ RR:

$$\begin{aligned} \text{VE} = 1 - \text{RR} &= 1 - \frac{p_v}{p_u} = 1 - \frac{d_v/n_v}{d_u/n_u} \\ \simeq 1 - \text{OR} &= 1 - \frac{\text{odds}_v}{\text{odds}_u} = 1 - \frac{d_v/h_v}{d_u/h_u}, \end{aligned} \tag{S2}$$

where p_v and p_u are the probabilities of disease (or risks) in vaccinated and unvaccinated groups, d_v and d_u are the respective numbers of observed cases. n_v and n_u are the population in the two groups, so $n = u + h$ with h_v and h_u are the controls, healthy individuals that did not experienced the disease or any other event of interest. The odds are defined as

$$\begin{aligned} \text{odds}_\text{v} &= \frac{p_v}{1 - p_v} \quad \text{and} \\ \text{odds}_\text{u} &= \frac{p_u}{1 - p_u}. \end{aligned} \tag{S3}$$

A variant of the case-control studies is the screening method [3]. As in case-control studies, it requires to compare the proportion of vaccinated among the cases and the control population. However it differs from other retrospective methods since control is achieved through external data: the vaccine coverage is estimated from a source that is external to the study, that has to be highly accurate.

Let's imagine to estimate the vaccine effectiveness by directly comparing the proportion of cases occurred in the vaccinated group (PCV) with the proportion of the total population that has been vaccinated (PPV). Then, Equation S2 can be rewritten as [3, 4]:

$$\text{VE} = 1 - \frac{\text{PCV}}{1 - \text{PCV}} \frac{1 - \text{PCV}}{\text{PPV}} \tag{S4}$$

Instead of drawing a sample of the population and estimating PPV from it, as in case-control studies, the screening method employs the assumed true value of PPV from monitoring systems.

Employing disease occurrence data, it is not possible to detect indirect effects of vaccines. An indirect effect would reduce the number of disease cases in all the population, independently on they vaccinal status. Therefore, PCV, that is a number of cases in vaccinated divided by the overall number of cases, would remain unchanged, and so would the VE. Moreover, as the indirect effectiveness increases, the samples of observed cases decrease, making the VE estimation always more difficult and even impossible, in the extreme case of perfect immunization against transmission that would imply null samples sizes.

	VE_{true}				
PPV	50%	60%	70%	80%	90%
	1717	1107	652	330	120
70%	474	309	185	95	35
	232	153	92	48	18
	1967	1205	664	307	98
80%	555	346	195	93	31
	277	175	100	49	17
	3022	1742	882	361	94
90%	874	516	271	117	33
	445	269	145	65	20
	5302	2950	1420	535	120
95%	1554	892	449	182	47
	801	471	246	105	30

Table S1: Number of cases to be ascertained for various values of VE_{true} and PPV, for three different given values of the 95% CI's lower half width VE_{true}-VE_{low}: 5% (first line in each cell), 10% (second line), 15% (third line).

S1.1 Screening method's sample size analysis

Estimates of vaccine effectiveness must be accompanied by a measure of their precision (or error), usually expressed as a 95% confidence interval (CI). The precision increases as the study sample increases. When designing effectiveness studies, it is crucial to calculate the sample size of cases needed to achieve a desired precision.

For the screening method, Farrington [4] derived a formula to calculate the number of cases x to be observed to estimate a hypothetical true value of VE_{true} with a lower 95% confidence limit above a certain threshold VE_{low}, depending on the proportion of the population that has been vaccinated (PPV):

$$x = \frac{1.96^2(1 - PPV \cdot VE_{low})^2(1 - VE_{true})}{PPV(1 - PPV)(VE_{true} - VE_{low})^2} \tag{S5}$$

Table S1 shows the sample sizes x calculated for different values of PPV, VE_{true} and the distance (VE_{true}-VE_{low}) of lower 95% confidence limit from the true effectiveness.

S1.2 The case of Bexsero

It is convenient to convert sample sizes into observational times, by employing the disease incidence observed in the past in the cohorts of interest. Table S2 reports the time, expressed in years, required to observe a number of MenB disease cases large enough to

	VE_{true}				
PPV	50%	60%	70%	80%	90%
70%	37.6<br>10.4<br>5.1	27.2<br>7.6<br>3.7	18.2<br>5.2<br>2.6	10.7<br>3.1<br>1.6	4.6<br>1.4<br>0.7
80%	46.7<br>13.2<br>6.6	33.0<br>9.5<br>4.8	21.5<br>6.3<br>3.3	12.2<br>3.7<br>1.9	5.0<br>1.6<br>0.9
90%	78.3<br>22.6<br>11.5	54.0<br>16.0<br>8.3	34.0<br>10.4<br>5.6	18.4<br>5.9<br>3.3	7.1<br>2.5<br>1.5
95%	143.9<br>42.2<br>21.7	97.8<br>29.5<br>15.6	60.4<br>19.1<br>10.5	31.8<br>10.8<br>6.2	11.8<br>4.6<br>2.9

Table S2: Expected years needed to observe an adequate sample size of MenB disease cases for various values of VE_{true} and PPV, for three different given values of the 95% CI's lower half width VE_{true}-VE_{low}: 5% (first line in each cell), 10% (second line), 15% (third line). Observational time is calculated using formula S9.

estimate VE_{true} with a lower 95% confidence limit VE_{low} lying 5%, 10% and 15% below VE_{true}, for different values of PPV.

In order to convert sample sizes into observational times, we employed recent reported incidences, assuming that future incidence will be the same if no vaccination is implemented. The purpose is to provide a conservative estimate of the observational time as the MenB disease cases have occurred with a decreasing trend during the last years. The total number of MenB disease cases reported during the last three epidemiological years for which these numbers are publicly available, i.e. 2011/12, 2012/13 and 2013/14, were 587, 595 and 424, respectively [5], corresponding to an average 535.3 cases per year. The effectiveness study will take into account only infants of one year of age, belonging to the fully vaccinated cohort. Public Health England does not provide data with such a fine age-stratification. Thus, we estimated the number of cases $z_{a=1}^{\text{past}}$ occurred in the age class of interest by multiplying the mid 2011 to mid 2014 average of the yearly total number of cases, k, times the normalised distributions of MenB cases by age observed in 2007 and in 2008, here denoted with $y_{a=1}$ (data available from MATS databases [6] and stratified by single year):

$$z_{a=1}^{\text{past}} = k \cdot y. \qquad \text{(S6)}$$

It resulted that $z_{a=1}^{\text{past}} = 70.2$ cases per year.

Let's now assume that, *if no vaccination is implemented*, during the 2015/16 and the following epidemiological years, the expected number of MenB disease cases in infants of 1 year of age will be:

$$z_{a=1}^{\text{no vaccine}} = z_{a=1}^{\text{past}}. \tag{S7}$$

In this case, it is possible to calculate also an expected yearly number of cases $z_{a=1}$ that will be reported *in case of vaccination*, providing hypothetical values of VE_{true} and PPV:

$$z_{a=1} = z_{a=1}^{\text{no vaccine}}(1 - \text{PPV} + \text{PPV}(1 - \text{VE}_{\text{true}})). \tag{S8}$$

Diving the sample size x calculated by fixing VE_{true}, VE_{low} and PPV (Equation S5) by the appropriate guess of number of cases $z_{a=1}$ expected during one year, it is finally possible to translate sample sizes in observational times T:

$$T = \frac{x}{z_{a=1}}. \tag{S9}$$

S2 Dynamic model for meningococcal carriage and disease

The dynamic model is structured into two parts:

1. a *transmission model* (Figure 1A in the main text), that, depending on the vaccine effectiveness values given as inputs, simulates the asymptomatic spreading of meningococcal carriage and the immunization campaign implementation in a specific population. The model output is the number of asymptomatic infection events.

2. An *observational model* (Figure 1B in the main text), that allows to build likelihoods given time series of IMD cases notified by public health authorities and the transmission model outcomes.

S2.1 Transmission model

The following notation is used to the define the compartments of the transmission model:

- S_a = susceptible unvaccinated of age class a,
- C_a = carriers unvaccinated of age class a,
- SVI_a = susceptible vaccinated and immune of age class a,
- CVI_a = carriers vaccinated and immune of age class a,
- SV_a = susceptible vaccinated but not immune of age class a,
- CV_a = carriers vaccinated but not immune of age class a.

Transition	Description	Parameter
$\otimes \to S_0$	birth	α
$X_a \to X_{a+1}$	ageing	–
$X_a \to \otimes$	death	μ_a
$S_a \to C_a$<br>$SV_a \to CV_a$	infection	$\lambda_a(t)$
$SVI_a \to CVI_a$	reduced infection	$\lambda_a(t) \cdot (1 - VE_{ind})$
$C_a \to S_a$<br>$CV_a \to SV_a$<br>$CVI_a \to SVI_a$	recovery	ρ
$S_a \to SVI$<br>$C_a \to CVI$	successful vaccination	$\gamma_a \cdot VE_{dir}$
$S_a \to SV$<br>$C_a \to CV$	unsuccessful vaccination	$\gamma_a \cdot (1 - VE_{dir})$
$SVI_a \to SV$<br>$CVI_a \to CV$	waning of immunity	ω_a and τ_{imm}

Table S3: Allowed transitions between compartments.

The population is divided into 160 age groups of three months size, plus one group that includes all individuals ≥ 40 years old. Thus, the index a ranges from 0 to 160.

Individuals are moved between compartments through three broad types of transitions: demographic, vaccinal and epidemiological. All the possible transitions are summarized in Table S3.

S2.1.1 Demographic transitions

The demographic transitions are births, ageing and deaths. Births consist in introducing new susceptible individuals into the first age class. Deaths remove individuals from all compartments, with a mortality rate μ_a that depends on the age class a. Ageing consists in moving *all* the subjects belonging to a compartment of a given age class to the same kind of compartment of the following age class, except for the last age class, from which subjects can only exit when they die.

S2.1.2 Vaccinal transitions

Vaccinal transitions (dashed arrows in Figure 1A in the main text) act according to vaccine schedules. Individuals are removed from compartments of unvaccinated according to the reported fractions of individuals covered by the campaign, namely γ_a. The precise time points t_{vac} at which individuals are moved depend on the vaccine and the vaccination

strategy. Individual can be moved only if they have received all the recommended doses and only after a time needed to enhance an immune response. Vaccinated individuals are divided into two classes: vaccinated immune and vaccinated non-immune. Here the *direct vaccine effectiveness* VE_{dir} comes into play. Only a fraction VE_{dir} of the vaccinated gains immunity to IMD, the remaining 1-VE_{dir} goes to non-immune compartments. However, after a certain time period, vaccines cease to be efficacious, so that vaccinated subjects lose their acquired immunity.

The waning of immunity is modelled by means of transitions SVI→SV and CVI→CV. Such transitions are activated after a time span τ_{imm} following the precise time of vaccination t_{vac}. The duration of immunity distributions are usually right-skewed, i.e. a fraction of subjects remains immune for a longer time if compared to the average. To take into account this feature, we model a fraction ω_a of immune individuals that lose their immunity after a time τ_{imm}, so that the remaining $1-\omega$ fraction of the immune individuals have a long-lasting immunity.

S2.1.3 Epidemiological transitions

The epidemiological transitions (infection and recovery) are responsible for the transmission of meningococcal carriage in the population. These transitions are stochastic in order to incorporate intrinsic noise. Therefore, the corresponding parameters can be interpreted as probabilities. They are obtained from rates, but the model's time scale has been chosen so that rates are a good approximation of probabilities [7, 1]. Specifically, all the stochastic processes are modelled as binomial processes.

All the carriers have the same chance ρ of recovering, regardless of their age and vaccinal state. If not immune, susceptibles can become carriers with a probability λ_a. Individuals in SVI compartments are instead subjected to a probability of becoming carriers reduced by a factor VE_{ind}, if compared to individuals in S and SV of the same age. The force of infection λ_a is defined as:

$$\lambda_a(t) = \beta_a \sum_{a'} m_{aa'} \frac{\mathrm{C}_{a'}(t) + \mathrm{CVI}_{a'}(t) + \mathrm{CV}_{a'}(t)}{N_{a'}(t)}, \tag{S10}$$

where $N_a(t) = \mathrm{C}_a(t) + \mathrm{CVI}_a(t) + \mathrm{CV}_a(t) + \mathrm{S}_a(t) + \mathrm{SVI}_a(t) + \mathrm{SV}_a(t)$ is the total population. The matrix elements $m_{a,a'}$ represent the number of contacts a subject of age a has with individuals of age a' during a time-step. Multiplied by the prevalence of carriage by age, it provides the pattern of potentially infectious contacts. This is then rescaled by β_a, that incorporates an age-dependent risk of meningococcal transmission and nasopharingeal colonization given a contact with an infected person.

At each time step, the number of individuals at risk of developing invasive disease, namely subjected to transitions $\mathrm{S}_a \to \mathrm{C}_a$ and $\mathrm{SV}_a \to \mathrm{CV}_a$, are counted. These counts are depicted in Figure 1A of the main text by the red swollen arrows. They are denoted with J_a for non-vaccinated, and JV_a for vaccinated not immune. The Js and the JVs are the

transmission model's *outcomes*, to be connected to the time series of *laboratory confirmed reported* IMD cases, that are the system's *observable*.

The progression to invasive meningococcal disease is not part of the transmission model. In fact, its contribution to the spreading of meningococcal carriage is negligible, since it affects a very small fraction of the susceptible S and SV that have been infected. The risk of IMD given infection will be instead used in the observational model.

S2.2 Observational model

The observational model described in the main text (Figure 1, panel B) highlights the link between the transmission model outcomes J_a and JV_a and the time series of IMD cases by age and vaccination status to be collected from the monitoring systems, such as Public Health England. We explicitly modelled an age-dependent probability θ_a of observing an IMD case given an underlying, not directly observable (but reproducible through the transmission model) infection event. We assumed no under-reporting, since IMD cases are so severe that is quite unlikely that they go unnoticed. For what concerns the laboratory confirmation (included serogroup ascertainment), we assumed that there is no bias across ages in this process.

The progression-to-disease process is modelled through a binomial distribution with parameters J (JV for the vaccinated non-immune) and θ. Therefore the number of IMD cases of age a observed during a certain time τ are distributed as :

$$D_a^{obs}(\tau) \simeq Bin(J_a(\tau), \theta_a), \tag{S11}$$

$$DV_a^{obs}(\tau) \simeq Bin(JV_a(\tau), \theta_a). \tag{S12}$$

θ_a is an age-dependet risk of disease given infection multiplied by a probability of laboratory confirmation and by a probability of reporting. Since we assume a perfect reporting, the latter equals 1. The probability of laboratory confirmation is a constant, whose precise value is not relevant to our aim. Therefore the θs can be called *risks of disease given infection*, but implying that they include also a probability of laboratory confirmation.

S2.3 Transmission model's equations

The equations governing the model dynamics are many and non-linear. Here we provide the deterministic skeleton of the transmission model's system of equations.

Equation without vaccinal transitions. When $a = 0$ (note that there are no carrier newborns):

$$\mathrm{N}_0(t+1) = (1-\mu_0)\alpha \sum_a \mathrm{N}_a(t) \tag{S13}$$

$$\mathrm{C}_0(t+1) = (1-\mu_0)\lambda_0(t)\alpha \sum_a \mathrm{N}_a(t). \tag{S14}$$

When $0 < a < 160$:

$$\begin{aligned} N_a(t+1) =& (1-\mu_a)N_{a-1}(t) \quad \text{(S15)} \\ C_a(t+1) =& (1-\mu_a)[C_{a-1}(t) \\ & + \lambda_a(t)(N_{a-1}(t) - C_{a-1}(t)) - \rho C_{a-1}(t)]. \quad \text{(S16)} \end{aligned}$$

When $a = 160$:

$$\begin{aligned} N_a(t+1) =& (1-\mu_a)[N_a(t) + N_{a-1}(t)] \quad \text{(S17)} \\ C_a(t+1) =& (1-\mu_a)[C_a(t) + C_{a-1}(t)+ \\ & + \lambda_a(t)(N_a(t) + N_{a-1}(t) - C_a(t) - C_{a-1}(t)) \\ & - \rho(C_a(t) + C_{a-1}(t))]. \quad \text{(S18)} \end{aligned}$$

$N_a = S_a + C_a$ is the total population of age a, while λ_a depends on two additional parameters that allow to model the meningococcal transmission patterns:

$$\lambda = \beta_a \sum_{a'} m_{aa'} \frac{C_{a'}(t)}{N_{a'}(t)}. \quad \text{(S19)}$$

β_a is a rate of meningococcal transmission and colonization given contact, while $m_{a,a'}$ is the number of contacts that an indicidual of age a has with an individual of age a' during a time-step.

Complete system of equations. When $a = 0$ (note that there are no carrier newborns, nor vaccinated newborns):

$$N_0(t+1) = (1-\mu_0)(1-\gamma_0(t))\alpha \sum_a N_a(t) \quad \text{(S20)}$$

$$NVI_0(t+1) = (1-\mu_0)(1-\omega_0(t))[\gamma_0(t) VE_{dir} \alpha \sum_a N_a(t)] \quad \text{(S21)}$$

$$NV_0(t+1) = \frac{\omega_0(t)}{1-\omega_0(t)} NVI_0(t+1) + (1-\mu_0)[\gamma_0(t)(1-VE_{dir})\alpha \sum_a N_a(t)] \quad \text{(S22)}$$

$$C_0(t+1) = (1-\mu_0)(1-\gamma_0(t))\lambda_0(t)\alpha \sum_a N_a(t) \quad \text{(S23)}$$

$$CVI_0(t+1) = (1-\mu_0)(1-\omega_a(t))\gamma_0(t) VE_{dir}(1-VE_{ind})\lambda_0(t)\alpha \sum_a N_a(t) \quad \text{(S24)}$$

$$CV_0(t+1) = \frac{\omega_0(t)}{1-\omega_0(t)} CVI_0(t+1)(1-\mu_0)\gamma_0(t)(1-VE_{dir})\lambda_0(t)\alpha \sum_a N_a(t) \quad \text{(S25)}$$

When $0 < a < 160$:

$$\mathrm{N}_a(t+1) = (1-\mu_a)(1-\gamma_a(t))\mathrm{N}_{a-1}(t) \tag{S26}$$

$$\mathrm{NVI}_a(t+1) = (1-\mu_a)(1-\omega_a(t))[\mathrm{NVI}_{a-1}(t) + \gamma_a(t)\mathrm{VE}_{dir}\mathrm{N}_{a-1}(t)] \tag{S27}$$

$$\mathrm{NV}_a(t+1) = \frac{\omega_a(t)}{1-\omega_a(t)}\mathrm{NVI}_a(t+1)(1-\mu_a)[\mathrm{NV}_{a-1}(t) + \gamma_a(t)(1-\mathrm{VE}_{dir})\mathrm{N}_{a-1}(t)] \tag{S28}$$

$$\begin{aligned}\mathrm{C}_a(t+1) = &(1-\mu_a)[(1-\gamma_a(t))\mathrm{C}_{a-1}(t)\\ &+ \lambda_a(t)(\mathrm{N}_{a-1}(t) - (1-\gamma_a(t))\mathrm{C}_{a-1}(t))\\ &- \rho(1-\gamma_a(t))\mathrm{C}_{a-1}(t)]\end{aligned} \tag{S29}$$

$$\begin{aligned}\mathrm{CVI}_a(t+1) = &(1-\mu_a)(1-\omega_a(t))[\mathrm{CVI}_{a-1} + \gamma_a(t)\mathrm{VE}_{dir}\mathrm{C}_{a-1}(t)\\ &+ \lambda_a(t)(1-\mathrm{VE}_{ind})(\mathrm{NVI}_{a-1}(t) - \mathrm{CVI}_{a-1}(t) - \gamma_a(t)\mathrm{VE}_{dir}\mathrm{C}_{a-1}(t))\\ &- \rho(\mathrm{CVI}_{a-1}(t) + \gamma_a(t)\mathrm{VE}_{dir}\mathrm{C}_{a-1}(t))]\end{aligned} \tag{S30}$$

$$\begin{aligned}\mathrm{CV}_a(t+1) = &\frac{\omega_a(t)}{1-\omega_a(t)}\mathrm{NVI}_a(t+1)(1-\mu_a)[\mathrm{CV}_{a-1} + \gamma_a(t)(1-\mathrm{VE}_{dir})\mathrm{C}_{a-1}(t)\\ &+ \lambda_a(t)(\mathrm{NV}_{a-1}(t) - \mathrm{CV}_{a-1}(t) - \gamma_a(t)(1-\mathrm{VE}_{dir})\mathrm{C}_{a-1}(t))\\ &- \rho(\mathrm{CV}_{a-1}(t) + \gamma_a(t)(1-\mathrm{VE}_{dir})\mathrm{C}_{a-1}(t))]\end{aligned} \tag{S31}$$

The complete set of deterministic equations governing the compartments of the last age class are not shown, since they are very verbose, but they can be easily derived from the above equations, as done for equation S17 and S18 . Here, $\mathrm{N}_a = \mathrm{S}_a + \mathrm{C}_a$, $\mathrm{NVI}_a = \mathrm{SVI}_a + \mathrm{CVI}_a$, and $\mathrm{NV}_a = \mathrm{SV}_a + \mathrm{CV}_a$. As displayed in the main text, $\lambda_a(t)$ is defined as:

$$\lambda_a(t) = \beta_a \sum_{a'} m_{aa'} \frac{\mathrm{C}_{a'}(t) + \mathrm{CVI}_{a'}(t) + \mathrm{CV}_{a'}(t)}{\mathrm{N}_{a'}(t) + \mathrm{NVI}_{a'}(t) + \mathrm{NV}_{a'}(t)}, \tag{S32}$$

with the same β_a and $m_{a,a'}$ defined after equation S19.

Note that in this deterministic formulation, here provided for illustrative purposes, the compartment populations and the numbers of cases are not integer numbers. Differently, in the stochastic model that we used, these numbers are naturally discrete when sampled from binomial distributions, or are forced to be discrete by rounding when they emerge from deterministic multiplications, as in the demographic transitions.

S2.4 Observational model's equations

It is possible to add to the previous systems of difference equations also the deterministic version of the observational model:

$$\begin{aligned} D_a(t) &= \theta_a J_a(t),\\ DV_a(t) &= \theta_a JV_a(t),\end{aligned} \tag{S33}$$

with D and DV number of IMD cases emerging from the numbers of meningococcal transmission events J and JV counted during the time-step t.

S3 Model parametrisation

The meningococcal model is a piece of an inferential framework to estimate the effectiveness of a vaccine in the field, once an immunisation campaign is introduced. Given the quantitative nature of the objective, the particular setting of the immunisation campaign is of primary importance. The case studies on which the model has been parametrised are two:

1. The serogroup C meningococcal vaccine introduction in England, started in November 1999;
2. The serogroup B meningococcal vaccine introduction in England, started in September 2015.

Even if the two vaccines under study act in immunologically very different ways, from the point of view of the model they are analogous. Differences lie in the precise values assigned to parameters and initial populations assigned to the compartments, that can be determined by the available data. The two implementations of the model will be hereafter sometimes referred to as *MenC model* and *MenB model*.

S3.1 Initial compartments

First of all, the compartments must be filled with the appropriate numbers of individuals, i.e. initial conditions have to be set. This task was accomplished using three ingredients:

- the England's population by year of age estimated at the time point of interest, immediately before the campaign start;
- the average meningococcal carriage prevalence by year of age;
- the fractions of carriers of serogroup B and serogroup C.

Population estimates are available from the UK Office of National Statistics [8] but they have a minimum resolution of one year. In order to fit the model's age classes, each 1-year-wide sub-population up to 39 years old is divided by four, while all the subjects older than 40 are aggregated into the last age class. The time points chosen for calibration are mid 1999 for the MenC model and mid 2014 for the MenB model.

The age pattern of carriage prevalence that we employ comes from of a recent meta-analysis, that merged several meningococcal carriage studies in Europe undertaken since the '70s [9]. As for the population, also the carriage prevalence has one year of resolution, so it is opportunely fitted to the age classes through cubic interpolation. The interpolation is done by fixing a boundary condition of null prevalence for negative ages.

The meta-analysis does not differentiate between serogroups, so it is rescaled using another source of data [10], that reported the results of three large large carriage studies,

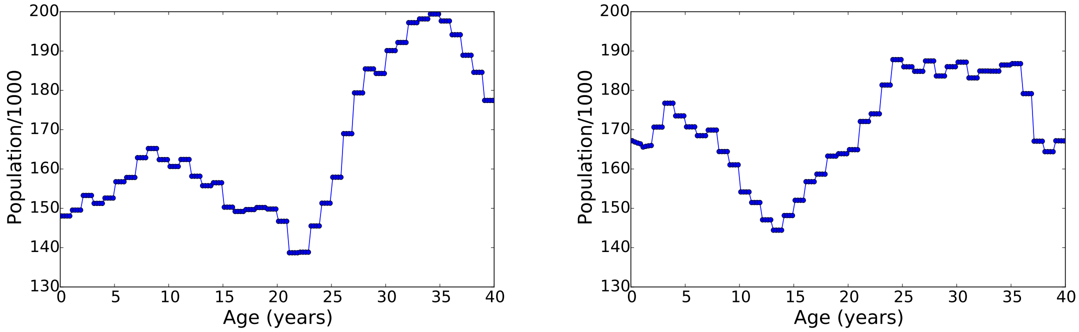

Figure S1: England's population by age up to 39 years of age, mid 1999 (left panel) and mid 2014 (right panel) estimates. The last age classes, composed by subjects of $\geq$40 years of age (not shown), count respectively 22.56 and 27.16 millions of individuals [8].

carried out in 1999, 2000, and 2001 in the UK, to determine the relative fractions of serogroup B and C.

Thus, the initial populations belonging to the compartments are:

$$\mathrm{C}_a = N_a c_a f_{serogroup}, \tag{S34}$$

$$\mathrm{S}_a = N_a - C_a, \tag{S35}$$

$$\mathrm{SVI}_a = \mathrm{CVI}_a = \mathrm{SV}_a = \mathrm{CV}_a = 0. \tag{S36}$$

N_a represents the population, already arranged by age classes of 3 months of width, c_a is the overall meningococcal carriage prevalence by age, and $f_{serogroup}$ is the fraction of serogroup B or serogroup C carriers. Their specific values are f_{MenB} =24.1% (the 2001 survey) and f_{MenC} =2.51% (1999 survey). Figure S1 shows the population distributions employed for the two case studies.

S3.2 Parametrisation of the transitions

Once the initial conditions are fixed, we set the parameters that regulate the transitions between compartments. Some of these quantities are known in literature, for instance demographic rates. In other cases, some calculations or additional assumptions are required to determine unknown quantities such the βs or the θs. A summary of the quantities needed to parameterise the model is shown in the main text, Table 1.

Births and deaths

The number of susceptible newborns introduced in the model at each quarter of year are generated by multiplying the crude birthrate α by the total population. α was obtained by

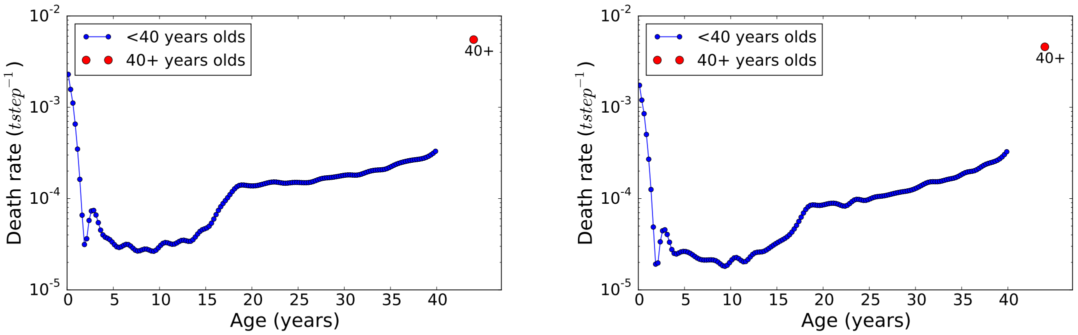

Figure S2: Mortality rates per 3 months time step, in England. Both are derived from notified deaths, averaged over two following years: 1999-2000 (left panel, used in the MenC model) and 2012-2013 (right panel, MenB model) [8]. y axes in log-scale.

dividing by four the yearly crude birthrates estimated in the years of interest in England and Wales [8]. For the MenB model, α is fixed to the most recent report, that is 12.3 births per 1000 population in 2013.

The mortality rates μ_a are derived from the notified deaths by age in England [8], that are firstly divided by the population, and then linearly interpolated to fit the model's age classes. Figure S2 shows the resulting mortality rates, plotted in logarithmic scale.

Recovery rate

The recovery rate ρ is taken as the reciprocal of the average duration of carriage τ_{car}, so that

$$\rho = 1/\tau_{car}. \tag{S37}$$

There are only a few longitudinal studies reporting the duration of meningococcal carriage, a quantity not easily obtainable and subject to large fluctuations. Median values reported in literature range between 4.1 and 10.2 months [11, 12, 13, 14] and no significant differences across age, serogroup and vaccination status are known. During the last years, modellers have mainly used a τ_{car} of six months for MenB [15, 16]. 6 months correspond to exactly 2 time steps of the model, thus $\rho = 0.5$ per time step. 6 months is here taken as the base case for duration of carriage, but other values of τ_{car} ranging between 4 and 10 months have been tested, as a sensitivity analysis (see section S5).

Infection rates

The λs regulate the infection transitions, but they depend on two other parameters (see equation 1 in the main text): the contact rates, m, and the rate of infection given contacts

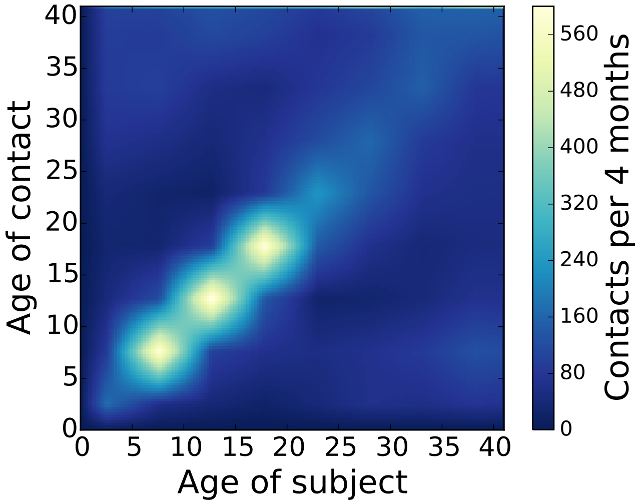

Figure S3: Contact patterns by age. Average number of different individuals of a certain age a' with which a single subject of a given age a daily establishes a contact, summed over three months. Darker colors represent lower numbers of contacts, lighter colors higher number of contacts.

with carriers, β.

The $m_{aa'}$ parameters correspond to the number of persons belonging to the age class a', that one person of age a is in contact with, during three months (one time step) in England. They are rearranged from the contact rates ϕ_{ij} surveyed in Great Britain during year 2005 (physical and non-physical contacts) [17]. Such contact rates are stratified to form 15 age classes (14 classes having 5 years of width, from 0-4 to 65-69 years, plus a 70+ years class), and they are *daily* contacts.

To get the m matrix, the ϕ matrix has been: (1) multiplied by the 2005 population in England arranged in the ϕ's age classes, to get the total number of contacts between ages; (2) grouped by ages higher or equal to 40 years; (3) divided by the 2005 population in England, also grouped for 40+ years old, to get back the number of contacts per single subject; (4) temporally rescaled to get contacts per time step of three months; (5) arranged in the a classes of the model, using a two-dimensional linear interpolation technique. The interpolation is done by fixing the boundary condition of null contacts for negative ages. The resulting contact pattern is shown in Figure S3.

The meningococcal asymptomatic transmission (or acquisition) given contact rates by age, that we called β_a, are still unknown in the literature. In order to parametrize them,

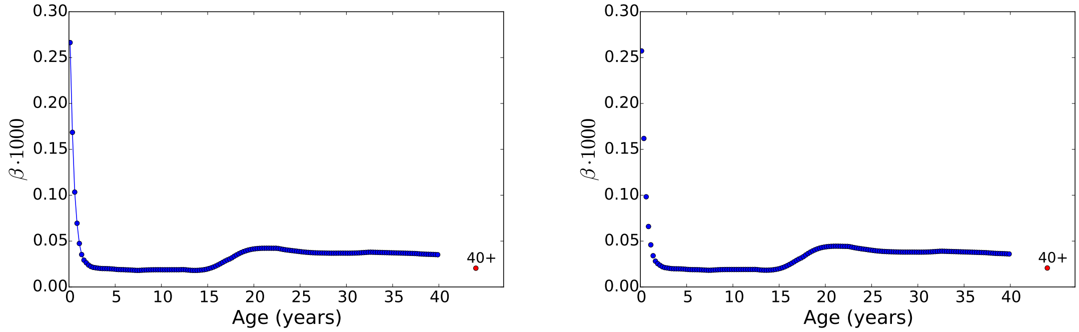

Figure S4: Values of β in the MenC model (left panel) and in the MenB model (right panel), calculated from equations S39 and S40.

we imposed the time-stability of the carriage prevalence, adding the following equation:

$$c_a(t+1) = c_a(t) \text{ for any } 0 \leq t < t_v, \tag{S38}$$

corresponding to a conservation of the prevalence $c_a = \mathrm{C}_a/N_a$ for each age a (N_a is the total population). Mathematically speaking, it consists in adding an equation containing no further variables that, acting as a constraint, allows to close the system of equations from S13 to S18. However, this assumption is not only a mere mathematical trick, but it is motivated by the fact that the prevalence of meningococcal carriage has a shape that is with a certain degree conserved in time [9]. Moreover, since our aim is building a tool for a *fast* inference of effectiveness, it seems reasonable to suppose that large fluctuations of the prevalence in short times (i.e. in less of 5 years) are unlikely, given the long duration of meningococcal carriage, and the slow dynamics of infection spreading. More detailed studies of meningococcal carriage would be precious in order to quantify transmission rates by age, or to verify and possibly modify our assumption.

After constraining equations from S13 to S18 with the condition S38, it is possible to calculate β_a. If a is not the last age ($a < 160$), for any time t,

$$\beta_a = \frac{N_{a-1}\frac{C_a}{N_a} - (1-\rho)C_{a-1}}{\sum_{a'} m_{aa'}\frac{C_{a'}}{N_{a'}}(N_{a-1} - C_{a-1})} \tag{S39}$$

where $\mathrm{N}_{-1} = \mathrm{N}_{\text{newborns}} = \alpha \sum_a N_a$ and $\mathrm{C}_{-1} = \mathrm{C}_{\text{newborns}} = 0$. For the last age class, $a = 160$,

$$\beta_a = \frac{(N_a + N_{a-1})\frac{C_a}{N_a} - (1-\rho)(C_a + C_{a-1})}{\sum_{a'} m_{aa'}\frac{C_{a'}}{N_{a'}}(N_a + N_{a-1} - C_a - C_{a-1})}\,. \tag{S40}$$

The values of β calculated with the above relationships in the MenC and the MenB settings are very similar (see Figure S4).

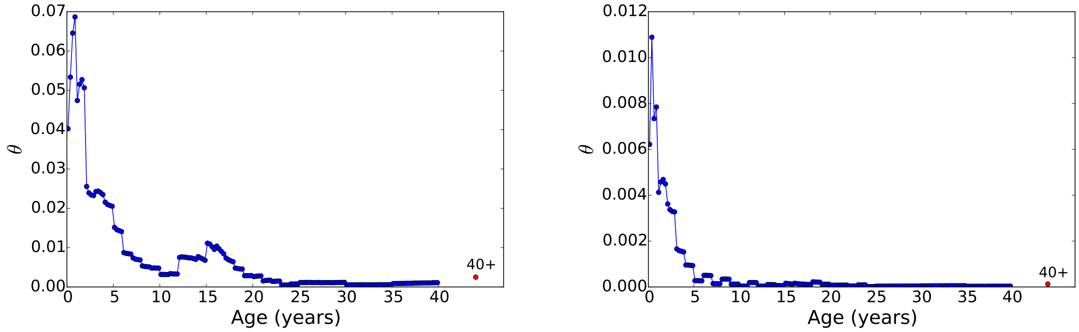

Figure S5: Values of θ in the MenC model (left panel) and in the MenB model (right panel), calculated employing equation S41.

Risks of disease given infection

The risk of disease given infection by age, θ_a, is calculated as

$$\theta_a = \frac{\mathrm{D}_a^{exp}}{J_a^{exp}}, \tag{S41}$$

where $\mathrm{D}_a^{exp}(t)$ is the expected occurrence of invasive disease during a time step if no vaccination is implemented, and $J_a^{exp}(t)$ is the number of expected infection events produced by the model during a time step if no vaccination is implemented. The calculation is based on the most recent available data before vaccination.

Specifically, for the MenC model D_a^{exp} was obtained by multiplying the normalised incidence of disease cases by age reported from January 1998 to June 1999 times the total number of cases observed in the epidemiological year 1998/1999, and then divided by 4 to get the desired time scale. However, we also tested different total numbers of cases (see section S5). For the MenB model the IMD incidence distribution employs cases reported in 2007 and 2008 in England and Wales, times the yearly average of the total number of cases reported in England from mid 2012 to mid 2015, then divided by 4 to get the desired time scale. The J^{exp} can be calculated using the infection term of the model equations, set at the initial time step:

$$J_a^{exp} = (1 - \mu_a)\lambda_a(0)(N_a(0) - C_a(0)). \tag{S42}$$

The θ rates do not depend on time, and can make sense only to reproduce meningococcal disease in regions where it is endemic, as in the UK: their calibration is based on the additional assumption that the the risk of developing invasive disease that susceptibles of a certain age will experience once infected will not change in the future, when other susceptibles of the same age will acquire the pathogen.

Vaccinal transitions

γ_a and ω_a are the parameters controlling the vaccination and the waning of immunity, respectively. They are modelled as on switches that, when activated, move a certain fixed fraction of a compartmental population.

For the MenB model, γ has in fact a single value, because the modelled immunisation campaign targets a single age class of infants, that are continuously targeted by a routine vaccination. The value of γ must be assumed because coverage data are not available yet. We assume here a coverage $\gamma = 95\%$ for the MenB vaccine implementation. Only children that have received all the three vaccine doses are considered vaccinated, therefore, during each time step, a fraction of unvaccinated individuals in the age class $a = 4$, corresponding to 12-14 months of age, are moved to the respective compartments of vaccinated.

The MenC immunisation campaign covered a much larger population. It has been composed by a large catch-up campaign, targeting individuals from 4 months to 18 years of age, extended later to cover up to 24 years old, plus a routine campaign designed with a 2+4+6 months schedule. In this case, coverage data are available in literature [18].

The fraction ω of immune individuals for which, after a certain while, the immunity wanes can be obtained from serological studies. Here we adopt the simplifying assumption that after a certain time τ_{imm} a fraction ω of the individuals belonging to a compartment of vaccinated immune is moved to a vaccinated non-immune compartment. This fraction is assumed to be $\omega = 100\%$ for the MenB vaccine, while the assumed duration of immunity is $\tau_{imm}^{MenB} = 18$ months after boost. For MenC, we used results of serological studies that estimated that a fraction $f_{imm} = 0.416$ of those who initially gained immunity was still immune 3-4 years after the last dose [19]. Therefore $\omega_a = 1 - 0.416 = 0.584$ for MenC. τ_{imm}^{MenC} was fixed to 15 months for routine vaccination, 5 years for catch-up of one year old infants, 10 years for all the others [20, 21].

S3.3 Sequential Monte Carlo

Both the VE are varied between 0 and 1 with a step of 0.001, so that the (VE_{dir}, VE_{ind}) space of the unknown parameters is a discrete grid. For each combination of the VEs, here we explain the process of likelihood building.

1. 1000 particles are generated. Each particle represents the variables of the systems, i.e. the populations belonging to each of the 966 compartmental populations. At the beginning, all the particles are set to the same compartments' initial conditions.

2. Then all the 1000 particles evolve for the first year y (4 model's time steps) in the transmission model. At the end of the process, they will have in general different values in the compartments' space, because of the stochasticity of the model. In addition to the updated state, each particle produces also an output: the Js by age and vaccination status.

3. The $J_{a,v}(y = 1)$ values are combined with the risks θ_a (both opportunely rescaled from age classes a to age groups b if needed) in the observational model, to calculate a binomial probabilities to observe $\mathrm{D}_{b,v}^{obs}(y = 1)$. These binomial probabilities multiplied together are the likelihood at $y = 1$. The particles are then *pruned* according to their specific likelihoods, using a weighted resampling algorithm [22], and, finally, the probabilities of the particles are averaged and a likelihood for the first year is obtained:

$$\prod_{b,v} P(\mathrm{D}_{b,v}^{obs}(y = 1)|\mathrm{VE}_{dir}, \mathrm{VE}_{ind}, H). \tag{S43}$$

Then, this procedure is repeated from point 2, substituting "first year" and "$y = 1$" with "second year" and "$y = 2$". The result is a second likelihood, conditional to the states and observations of the first year:

$$\prod_{b,v} P(\mathrm{D}_{b,v}^{obs}(y = 2)|\mathrm{D}_{b,v}^{obs}(y = 1), \mathrm{VE}_{dir}, \mathrm{VE}_{ind}, H). \tag{S44}$$

Going on for the following years, using the Markov property, all the conditional likelihoods are multiplied together to eventually get the likelihood shown in equation 3 of the main text. Once the final likelihood at year Y is calculated for the particular set of VEs chosen at the beginning, then the VEs are varied, a new set of 1000 particles is created to finally produce a likelihood for another point of the VE's grid. The procedure is repeated until all the (VE_{dir}, VE_{ind}) grid is explored. At the end, a surface like the one shown in Figure 1C of the main text is produced.

Technical notes: to run the SMC algorithm described above, we run multiple python scripts on computer clusters; instead of the likelihood, log-likelihood were almost always actually used, in order to transform products in sums and avoid computational underflow problems.

S3.4 Maximum Likelihood and confidence intervals

Once we generated the likelihood surface at a desired time point, it is straightforward to get the best estimates VE_{dir}^{best} and VE_{dir}^{best} from the maximum of the likelihood itself.

To calculate the 95% confidence intervals (CIs) around the best estimates, it is convenient to work with the logarithm of likelihood $l = log((L))$. The loglikelihood l is first sliced by fixing one of the VEs on the value that maximise the l, then using the Wilks theorem [23], the 95% confidence interval can be calculated to be approximately included in the region for which:

$$l > \max(l) - \frac{h}{2}, \tag{S45}$$

where h is a chi-squared-distributed variable with one degree of freedom, so that $h = 0.381$ with a confidence level of 0.05.

However, before applying the Wilks theorem, the l values must be smoothed, since they are affected by Monte Carlo variability [22]. Here this is accomplished by applying a Savitzky-Golay filter. Finally, since l depends on discrete values of VE, it is linearly interpolated to be continuous in all the VE values between 0 and 1.

Examples of sliced likelihoods produced with this method are shown in Figure 2B of the main text, where the vertical lines point out best estimate and the 95% CIs.

S4 The mathematical model realistically reproduces demography and meningococcal epidemiology in England

We developed a mathematical model to reproduce two different vaccination campaigns in England, against serogroup C and B meningococcal disease. In both cases, here we test the model running its deterministic version without vaccination campaigns for 50 years, to see if it reasonably reproduce empirical evidences.

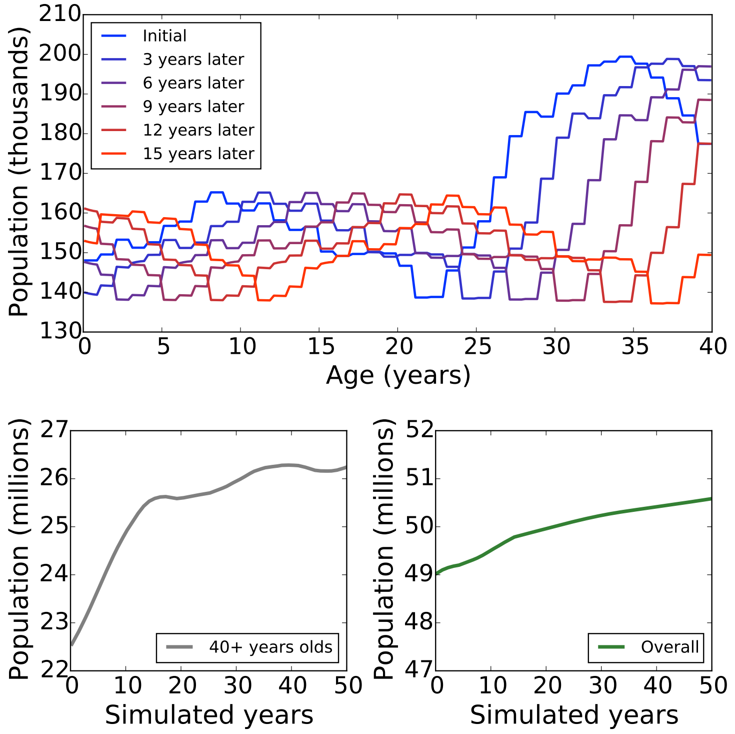

Figure S6: Evolution of the England's population surveyed in 1999, predicted by the MenC model.

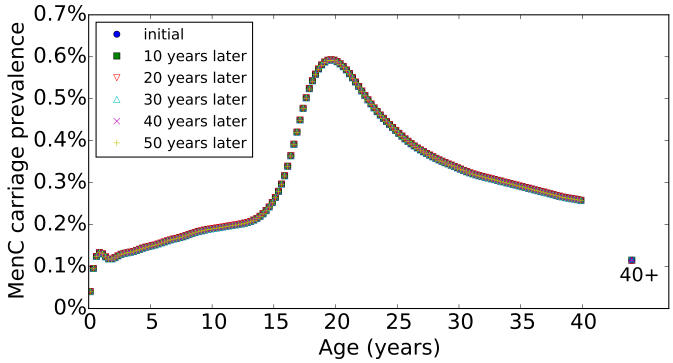

Figure S7: Evolution of MenC carriage prevalence in England predicted by the MenC model without vaccination.

The model reproduces a realistic demographic evolution of the population in England, with fluctuations due to population differences across age groups. Figure S6 shows the evolution of the demography for the MenC setting.

The carriage prevalence remains constant for all age groups for both MenB and MenC (MenC carriage shown in Figure S7), indicating that the model has been well calibrated under the assumption of temporal stability of the asymptomatic carriage prevalence.

We calculated the serogroup-specific transmission given contact rates β_a using equations S39 and S40, that basically depend on the following key quantities: the serogroup carriage prevalence by age and the duration of carriage. Direct estimates of transmission rates are never been published in literature, except for a recent work that evaluated a single carriage acquisition rate, for MenB only, in individuals of age between 10 and 25 years, finding 2.8 per 1000 person-month [24]. We tested whether our own acquisition rates are reasonably similar to the above clinical study's findings. To produce comparable numbers, we run the MenC and MenB models in absence of vaccination and then we simulated the statistical procedure adopted by the authors: we divided the overall number of transmissions J of the interested age classes by the starting susceptible population in the same age classes. We found the following acquisition rates: 1.32 per 1000 person-month in the MenC setting and 6.93 per 1000 person-moths in the MenB setting.

Figure S8 shows the distribution of MenC disease cases predicted without implementing any vaccination campaign. The cases have been numerically generated combining the disease equations S33 with the deterministic version of the transmission model, defined by equations from S13 to S18. The cases occurrences by age remain roughly constant, with fluctuations only due to the demographic shifts. An analogous behaviour is observed in the MenB setting (not shown). The disease endemicity is a direct consequence of the assumption that the risk of disease given transmission remains constant over time.

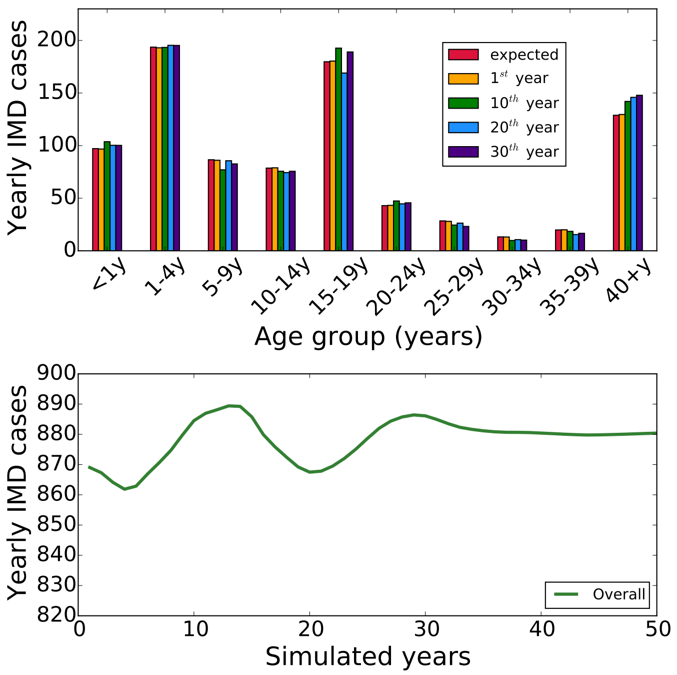

Figure S8: Evolution of MenC disease cases occurrences in England as predicted by the MenC model without vaccination. Simulated IMD cases by age are almost constant, with little fluctuations due to demography. These fluctuations flatten out after about 40 years (lower panel, overall incidence plotted against simulated years), since the model does not account for unpredictable external factors, such as migratory fluxes or drastic changes in birth and death rates.

For the MenC setting, it is also possible to check if the mathematical model correctly reproduces meningococcal epidemiology once the immunization campaign is implemented. In Figure 3 of the main manuscript, panel A, we showed that when the VE parameters are set to the MCML best estimates, the model well reproduces the overall number of reported cases per epidemiological year. Here we show that the same argument is valid looking at different age classes.

Figure S9 shows 6 panels, each one depicting time series of cases reported in England (grey bars) for 6 different age classes. The red points are the synthetic disease cases generated by our model set on our best estimates of VE_{dir} and VE_{ind}. In general, our simulation are in good agreement, demonstrating that the vaccination has been correctly modelled. However, the age group 20-29 yr shows a discrepancy: the real cases grew while instead of declining as predicted by our model. We note that such age class was not targeted by

the catch-up campaign in 2000/01. We hypothesize that some large environmental fluctuations, not reproducible by our model, brought to the increase of disease cases. Anyway, the next year the cases drastically fell, probably mainly for the herd immunity effect. It is possible that the mixing assumptions of our model are too simplistic or not very accurate. Maybe that individuals in the 20-29 age class have more contacts within themselves and less with the remaining population, so retarding the herd immunity effect. Further studies on human contacts and their impact on the transmission of *N. meningitidis* would be precious to improve the understanding and modelling of meningococcal disease.

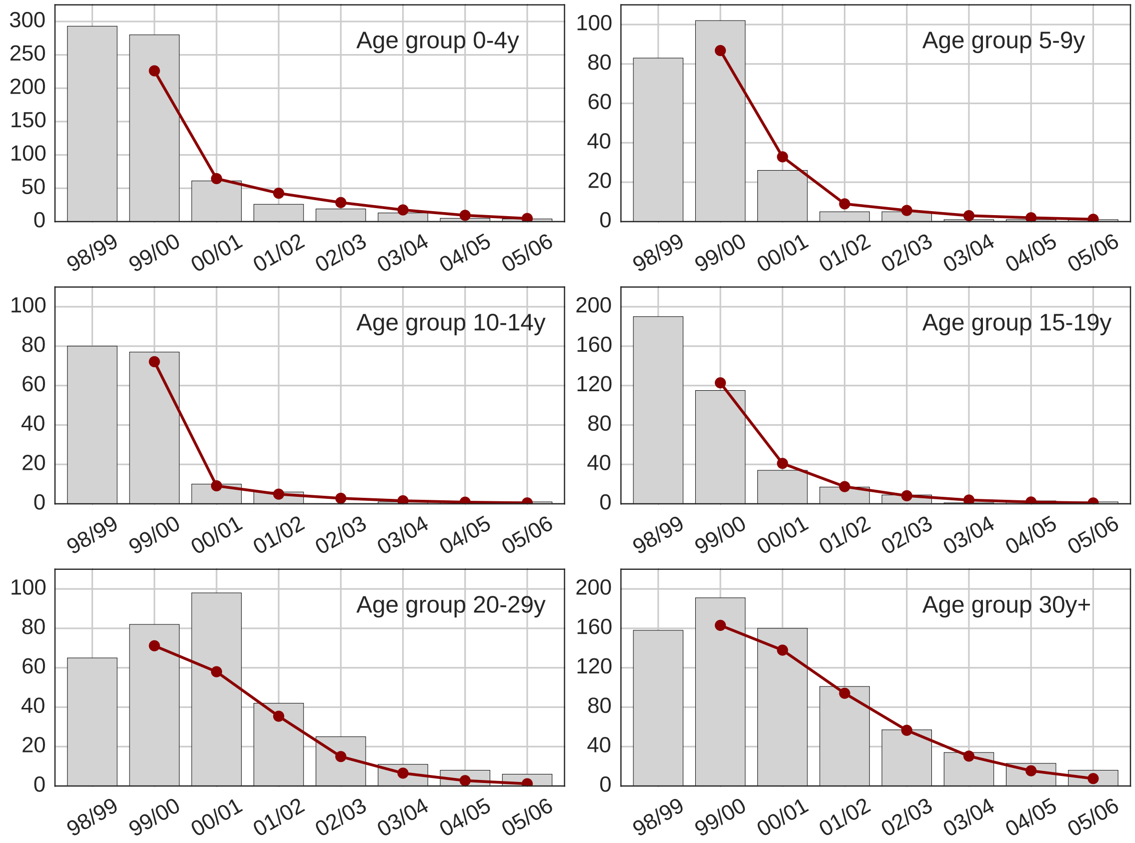

Figure S9: MenC epidemiology by age in England during the immunization campaign, started at the end of 1999. Each panel shows a different age group. Reported cases as grey bars, simulated cases as red dots. The simulations employed MCML best estimates of VE.

S5 Sensitivity analysis

The complexity of the inferential method and of the underlying mathematical meningococcal model required to test the sensitivity of the VE final estimates. We varied the expected number of disease cases, the duration of carriage and the duration of immunity. We tested these three key quantities because they are not accurately known and because all other quantities that are affected by uncertainties depend on them. Finally we discuss the temporal evolution of the final VE estimates. The sensitivity analyses were carried out in the MenC setting.

Expected number of disease cases

The number of disease cases by age can be subjected to unpredictable trends. We parametrized the risk of disease given infection (equation S41) using the average age-pattern of cases reported in England from January 1998 and June 1999, multiplied by total number of cases reported in England in epidemiological year 1998/99.

We tested whether different choices of the total number of cases, namely D_{tot}, would affect the MCML inference. In particular we set D_{tot} to be:

- England cases of known age in 98/99 (base case) = 869;
- England cases in 98/99 = 883;
- Yearly average England cases in 97/98[1] and 98/99 = 807;
- Yearly average England cases from 96/97[1] to 98/99 = 776;
- Yearly average England cases from 95/96[1] to 98/99 = 728;
- Low incidence scenario = 600;
- High incidence scenario = 1000.

Table S4 shows the final estimates of VE_{dir} and VE_{ind} and after two years of vaccination campaign, when D_{tot} is changed. The direct effectiveness is almost perfectly unchanged. The indirect effectiveness gets higher as the number of expected cases is increased.

Duration of carriage

Figure S10 shows how the likelihoods of VE_{dir} and VE_{ind} for the MenC vaccination setting change i) in time and ii) by varying the duration of carriage. Each row shows likelihoods

[1] We did not know the precise number of cases observed in England before 1998, therefore we had to use the aggregated England + Wales number, multiplied by the average fraction Eng over Eng&Wal MenC cases observed after 1998.

$\mathbf{D}_{tot}$	$\mathbf{VE}_{dir}$(%)	95%CI	$\mathbf{VE}_{ind}$(%)	95%CI
600	96.60	[94.82-97.89]	17.40	[5.51-31.06]
728	96.40	[94.69-97.86]	44.10	[30.64-57.72]
776	96.50	[94.74-97.88]	52.10	[38.92-66.52]
807	96.50	[94.74-97.85]	57.70	[44.18-72.26]
869 (baseline)	96.50	[94.76-97.86]	68.80	[54.43-83.27]
883	96.50	[94.77-97.86]	70.10	[56.39-85.37]
1000	96.60	[94.78-97.87]	89.10	[74.51-99.99]

Table S4: VE_{dir} and VE_{ind} best estimates and 95%CIs calculated through the MCML method (MenC setting). Each row corresponds to a different total number of disease cases D_{tot}, expected to emerge without vaccination.

calculated at different time points (from top to bottom, after 1 to 5 full epidemiological years from the campaign start). Each column corresponds to a different scenario of duration of carriage: from left to right, τ_{car} = 4, 6, 8 months, where 6 months is the base case supported by recent studies, as discussed in section S3.2. The likelihood presented in Figure 2 of the main text is shown here at the intersection of the second row from top (2 years of data) and second column (6 months of duration of carriage).

The MCML method is in general sensitive to the precise value assigned to the duration of carriage τ_{car}. However, only the indirect vaccine effectiveness is affected by changes to τ_{car}: moving from 4 to 8 months, the 95% confidence region shifts only horizontally. While VE_{dir} is fairly independent on τ_{car}, VE_{ind} is clearly affected. In the 4 months scenario, VE_{ind} converges to less than 50%, while if τ_{car} equals 8 months, VE_{ind} almost reaches 100%. When τ_{car} is set to 6 months, VE_{ind} converges to about 70%, a value very near to the ones calculated from carriage studies [25, 10], as described in main text.

Duration of immunity

We also tested the sensitivity of our results to the duration of immunity, τ_{imm}, whose value is reported in the literature with significant uncertainty. As described in the main text, in the MenC model we have set three different values, depending from the age of the vaccinated: 15 months for routinely vaccinated infants, 5 years for infants of up to 12 months of age enrolled for the catch-up campaign, 10 years for all the other subjects included in the catch-up campaign. Varying the duration of immunity induced in subjects vaccinated in the catch-up campaign does not change the results. This is a trivial consequence of the fact that we do not use disease case notifications after 5-6 years from the beginning of the campaign.

Regarding the routine vaccination, changing the duration of immunity slightly affects

our results. Figure S11 shows likelihoods of VE_{dir} and VE_{ind} for the MenC routine vaccination programme, calculated at different time points, by employing three different duration of immunity: 9 months, 15 months (base case [20, 21]) and 24 months. Differences in the best estimates are small. However, the 24 months scenario displays a higher value of the likelihood maximum, indicating that previous estimates could underestimate the duration of immunity. We think that the immunity persistence should be further investigate to improve the modelling of this process, that in other scenarios could significantly impact the VE estimation and Public Health decisions.

Temporal evolution of the VE estimates

In addition, Figures S10 and S11 also show how the estimates evolve in time (epidemiological years, overall between mid 2000 and mid 2005). In general, the 95% confidence regions shrink as more data are used. When IMD cases notified during the first epidemiological year only are employed, the best estimates of VE_{ind} are significantly different if compared to the best estimates obtained using more data, even if the precision after one year is very low, with a very broad 95% CI of the indirect effectiveness. On the other hand, the assessment of VE_{dir} is extremely fast to converge and does not show systematic deviations, indicating that the direct effectiveness of MenC conjugate vaccines in England could have been estimated even after one year only.

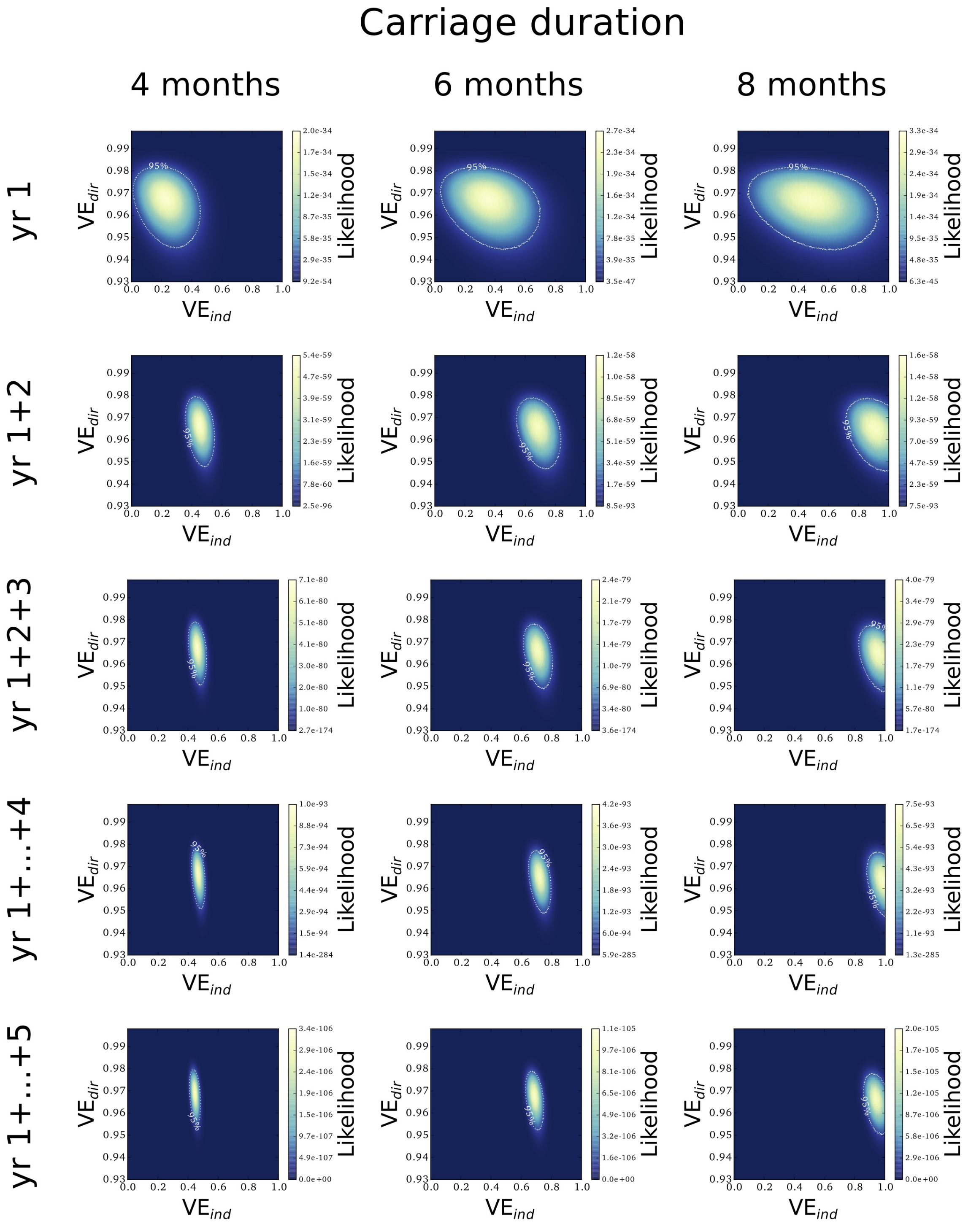

Figure S10: Likelihoods of VE_{dir} and VE_{ind}, MenC setting. Each column corresponds to a different value of the duration of carriage τ_{car}. Each row from top to bottom reports the likelihood obtained using data reported during 1, 2, 3, 4 and 5 epidemiological years after vaccination starts, i.e. between mid 2000 and at most mid 2005. Higher values of likelihood are shown in lighter colors. The 95% confidence regions are delimited by white dashed lines.

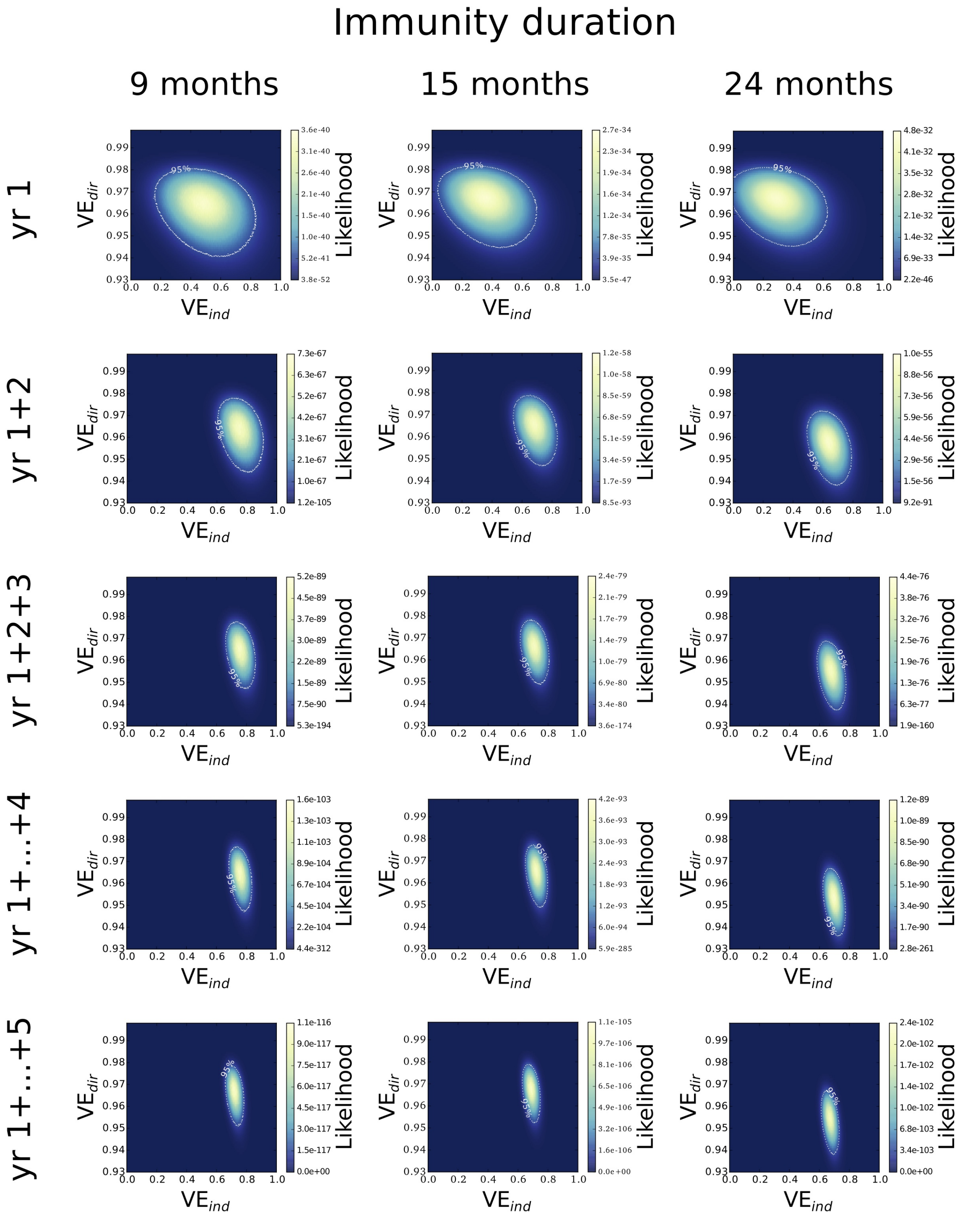

Figure S11: Likelihoods of VE$_{dir}$ and VE$_{ind}$, MenC setting. Each column corresponds to a different value of the duration of immunity τ_{imm} for routine vaccination. Each row from top to bottom reports the likelihood obtained using data reported during 1, 2, 3, 4 and 5 epidemiological years after vaccination starts, i.e. between mid 2000 and at most mid 2005. Higher values of likelihood are shown in lighter colors. The 95% confidence regions are delimited by white dashed lines.

References

[1] M Elizabeth Halloran, Ira M Longini Jr., and Claudio J Struchiner. *Design and Analysis of Vaccine Studies: Introduction.* Statistics for Biology and Health. Springer, 2009.

[2] James J Schlesselman and Paul D Stolley. *Case-Control Studies. Design, Conduct, Analysis.* Oxford University Press, 1982.

[3] W. a. Orenstein, R. H. Bernier, T. J. Dondero, a. R. Hinman, J. S. Marks, K. J. Bart, and B. Sirotkin. Field evaluation of vaccine efficacy. *Bulletin of the World Health Organization*, 63(6):1055–1068, 1985.

[4] C P Farrington. Estimation of vaccine effectiveness using the screening method. *International journal of epidemiology*, 22(4):742–746, aug 1993.

[5] Public Health England. Meningococcal disease: laboratory confirmed cases in England and Wales.

[6] Ulrich Vogel, Muhamed-Kheir Taha, Julio A Vazquez, Jamie Findlow, Heike Claus, Paola Stefanelli, Dominique A Caugant, Paula Kriz, Raquel Abad, Stefania Bambini, Anna Carannante, Ala Eddine Deghmane, Cecilia Fazio, Matthias Frosch, Giacomo Frosi, Stefanie Gilchrist, Marzia M Giuliani, Eva Hong, Morgan Ledroit, Pietro G Lovaglio, Jay Lucidarme, Martin Musilek, Alessandro Muzzi, Jan Oksnes, Fabio Rigat, Luca Orlandi, Maria Stella, Danielle Thompson, Mariagrazia Pizza, Rino Rappuoli, Davide Serruto, Maurizio Comanducci, Giuseppe Boccadifuoco, John J Donnelly, Duccio Medini, and Ray Borrow. Predicted strain coverage of a meningococcal multicomponent vaccine (4CMenB) in Europe: a qualitative and quantitative assessment. *The Lancet Infectious Diseases*, 13(5):416–425, may 2013.

[7] Duygu Balcan, Hao Hu, Bruno Goncalves, Paolo Bajardi, Chiara Poletto, Jose J Ramasco, Daniela Paolotti, Nicola Perra, Michele Tizzoni, Wouter Broeck, Vittoria Colizza, and Alessandro Vespignani. Seasonal transmission potential and activity peaks of the new influenza A(H1N1): a Monte Carlo likelihood analysis based on human mobility. *BMC Medicine*, 7(1):45, 2009.

[8] Office of National Statistics. Population.

[9] Hannah Christensen, Margaret May, Leah Bowen, Matthew Hickman, and Caroline L Trotter. Meningococcal carriage by age: a systematic review and meta-analysis. *The Lancet Infectious Diseases*, 10(12):853–861, dec 2010.

[10] Martin C. J. Maiden, Ana Belén Ibarz-Pavón, Rachel Urwin, Stephen J. Gray, Nicholas J. Andrews, Stuart C. Clarke, A. Mark Walker, Meirion R. Evans, J. Simon

Kroll, Keith R. Neal, Dlawer A A Ala'aldeen, Derrick W. Crook, Kathryn Cann, Sarah Harrison, Richard Cunningham, David Baxter, Edward Kaczmarski, Jenny Maclennan, J. Claire Cameron, and James M. Stuart. Impact of meningococcal serogroup C conjugate vaccines on carriage and herd immunity. *The Journal of infectious diseases*, 197(5):737–43, mar 2008.

[11] R Gold, I Goldschneider, M L Lepow, T F Draper, and M Randolph. Carriage of Neisseria meningitidis and Neisseria lactamica in infants and children. *The Journal of infectious diseases*, 137(2):112–121, feb 1978.

[12] Philippe De Wals and A Bouckaert. Methods for estimating the duration of bacterial carriage. *International journal of epidemiology*, 14(4):628–634, dec 1985.

[13] D A Caugant, C Fogg, F Bajunirwe, P Piola, R Twesigye, F Mutebi, L O Frøholm, E Rosenqvist, V Batwala, I S Aaberge, J A Rottingen, and P J Guerin. Pharyngeal carriage of Neisseria meningitidis in 2-19-year-old individuals in Uganda. *Trans R Soc Trop Med Hyg*, 100(12):1159–1163, 2006.

[14] I. C. Glitza, I. Ehrhard, B. Müller-Pebody, R. Reintjes, T. Breuer, a. Ammon, and H. G. Sonntag. Longitudinal study of meningococcal carrier rates in teenagers. *International Journal of Hygiene and Environmental Health*, 211(3-4):263–272, jul 2008.

[15] Hannah Christensen, Matthew Hickman, W. John Edmunds, and Caroline L. Trotter. Introducing vaccination against serogroup B meningococcal disease: An economic and mathematical modelling study of potential impact. *Vaccine*, 31(23):2638–2646, may 2013.

[16] H. Christensen, C. L. Trotter, M. Hickman, and W. J. Edmunds. Re-evaluating cost effectiveness of universal meningitis vaccination (Bexsero) in England: modelling study. *Bmj*, 349(oct09 4):g5725–g5725, 2014.

[17] Joël Mossong, Niel Hens, Mark Jit, Philippe Beutels, Kari Auranen, Rafael Mikolajczyk, Marco Massari, Stefania Salmaso, Gianpaolo Scalia Tomba, Jacco Wallinga, Janneke Heijne, Malgorzata Sadkowska-Todys, Magdalena Rosinska, and W. John Edmunds. Social Contacts and Mixing Patterns Relevant to the Spread of Infectious Diseases. *PLoS Medicine*, 5(3):e74, mar 2008.

[18] C L Trotter, M E Ramsay, and E B Kaczmarski. Meningococcal serogroup C conjugate vaccination in England and Wales: coverage and initial impact of the campaign. *Communicable disease and public health / PHLS*, 5(3):220–225, sep 2002.

[19] H. Campbell, N. Andrews, R. Borrow, C. Trotter, and E. Miller. Updated Postlicensure Surveillance of the Meningococcal C Conjugate Vaccine in England and Wales:

Effectiveness, Validation of Serological Correlates of Protection, and Modeling Predictions of the Duration of Herd Immunity. *Clinical and Vaccine Immunology*, 17(5):840–847, may 2010.

[20] Caroline L Trotter, Nick J Andrews, Edward B Kaczmarski, Elizabeth Miller, and Mary E Ramsay. Effectiveness of meningococcal serogroup C conjugate vaccine 4 years after introduction. *The Lancet*, 364(9431):365–367, 2004.

[21] C. L. Trotter. Dynamic Models of Meningococcal Carriage, Disease, and the Impact of Serogroup C Conjugate Vaccination. *American Journal of Epidemiology*, 162(1):89–100, jul 2005.

[22] E. L. Ionides, C. Breto, and A. A. King. Inference for nonlinear dynamical systems. *Proceedings of the National Academy of Sciences*, 103(49):18438–18443, dec 2006.

[23] J Th Van Der Kamp, Lisse Swets, and Gerhard H Fischer. *Statistical Models in.* 1985.

[24] Catherine A Jeppesen, Matthew D Snape, Hannah Robinson, Nicoletta Gossger, Tessa M John, Merryn Voysey, Shamez Ladhani, Ifeanyichukwu O Okike, Clarissa Oeser, Alison Kent, Jennifer Oliver, Pippa Taylor, Begonia Morales-Aza, Stuart C Clarke, Michelle Casey, Filipa Martins, Nicholas R E Kitchin, Annaliesa S Anderson, Hal Jones, Kathrin U Jansen, Joseph Eiden, Louise Pedneault, Paul T Heath, Adam Finn, Saul N Faust, and Andrew J Pollard. Meningococcal carriage in adolescents in the United Kingdom to inform timing of an adolescent vaccination strategy. *The Journal of infection*, 71(1):43–52, jul 2015.

[25] Martin CJ Maiden and James M Stuart. Carriage of serogroup C meningococci 1 year after meningococcal C conjugate polysaccharide vaccination. *The Lancet*, 359(9320):1829–1830, may 2002.
